# Impact of Donor Activating KIR Genes on HSCT Outcome in C1-Ligand Negative Myeloid Disease Patients Transplanted with Unrelated Donors—A Retrospective Study

**DOI:** 10.1371/journal.pone.0169512

**Published:** 2017-01-20

**Authors:** Christine Neuchel, Daniel Fürst, Dietger Niederwieser, Donald Bunjes, Chrysanthi Tsamadou, Gerald Wulf, Michael Pfreundschuh, Eva Wagner, Gernot Stuhler, Hermann Einsele, Hubert Schrezenmeier, Joannis Mytilineos

**Affiliations:** 1 Institute of Clinical Transfusion Medicine and Immunogenetics Ulm, German Red Cross Blood Transfusion Service, Baden Wuerttenberg–Hessen and University Hospital of Ulm, Ulm, Germany; 2 Institute of Transfusion Medicine, University of Ulm, Ulm, Germany; 3 Department of Hematology/Oncology, University of Leipzig, Leipzig, Germany; 4 Department of Hematology/Oncology, University Clinic Ulm, Ulm, Germany; 5 Department of Hematology/Oncology, Georg-August-University Göttingen, Göttingen, Germany; 6 Department of Internal Medicine I, Universitätsklinikum des Saarlandes, Homburg, Germany; 7 Department of Medicine III, Johannes Gutenberg-University Mainz, Mainz, Germany; 8 Centre for Bone Marrow and Blood Stem Cell Transplantation, Deutsche Klinik für Diagnostik, Wiesbaden, Germany; 9 Department of Medicine II, University Hospital Würzburg, Würzburg, Germany; 10 DRST–German Registry for Stem Cell Transplantation, Essen, Germany; University of Sydney, AUSTRALIA

## Abstract

Natural Killer cells (NK) are lymphocytes with the potential to recognize and lyse cells which escaped T-cell mediated lysis due to their aberrant HLA expression profiles. Killer cell immunoglobulin-like receptors (KIR) influence NK-cell activity by mediation of activating or inhibitory signals upon interaction with HLA-C (C1, C2) ligands. Therefore, absence of ligands for donor inhibitory KIRs following hematopoietic stem cell transplantation (HSCT) may have an influence on its outcome. Previous studies showed that C1 negative patients have a decreased HSCT outcome. Our study, based on a cohort of 200 C1-negative patients, confirmed these findings for the endpoints: overall survival (OS: HR = 1.41, CI = 1.14–1.74, p = 0.0012), disease free survival (DFS: HR = 1.27, CI = 1.05–1.53, p = 0.015), treatment related mortality (TRM: HR = 1.41, CI = 1.01–1.96, p = 0.04), and relapse incidence (RI: HR = 1.33, CI = 1.01–1.75, p = 0.04) all being inferior when compared to C1-positive patients (n = 1246). Subsequent analysis showed that these findings applied for patients with myeloid malignancies but not for patients with lymphoproliferative diseases (OS: myeloid: HR = 1.51, CI = 1.15–1.99, p = 0.003; lymphoblastic: HR = 1.26, CI = 0.91–1.75, p = 0.16; DFS: myeloid: HR = 1.31, CI = 1.01–1.70, p = 0.04; lymphoblastic: HR = 1.21, CI = 0.90–1.61, p = 0.21; RI: myeloid: HR = 1.31, CI = 1.01–1.70, p = 0.04; lymphoblastic: HR = 1.21, CI = 0.90–1.61, p = 0.21). Interestingly, within the C1-negative patient group, transplantation with KIR2DS2 resulted in better OS (9/10 matched: HR = 0.24, CI = 0.08–0.67, p = 0.007) as well as DFS (9/10 matched: HR = 0,26, CI = 0.11–0.60, p = 0.002), and transplantation with KIR2DS1 positive donors was associated with a decreased RI (HR = 0.30, CI = 0.13–0.69, p = 0.005). TRM was increased when the donor was positive for KIR2DS1 (HR = 2.61, CI = 1.26–5.41, p = 0.001). Our findings suggest that inclusion of KIR2DS1/2/5 and KIR3DS1-genotyping in the unrelated donor search algorithm of C1-ligand negative patients with myeloid malignancies may prove to be of clinical relevance.

## Introduction

Haematopoietic stem cell transplantation (HSCT) has been established as a potentially curative treatment for a variety of haematologic diseases. However, malignancy relapse after HSCT remains the most frequent cause of treatment failure[1]. Therefore, introduction of a T-cell mediated graft-versus-leukemia (GvL) effect is of great importance for a successful treatment[2]. Unfortunately, there are downsides to this T-cell alloreactivity as it is associated with graft-versus-host disease (GvHD)[3;4] which is a major complication following HSCT. Furthermore, transformed cells may escape T-cell mediated lysis by down regulation of the Human Leukocyte Antigen (HLA) expression on their cell surface[5–7]. On the other hand, Natural Killer cells (NK) are lymphocytes that have the potential to recognize and lyse cells with aberrant HLA-expression profiles, thereby mediating a GvL-effect of their own. It has been suggested that this competence qualifies NK cells to conduct an anti-leukemic immune response without causing a detrimental GvHD[8;9]. The functions of NK cells are controlled by interactions between NK cell receptors and ligand molecules on the respective target cells. NK cell targeting of leukemic cells is facilitated, among others, by the communication between killer cell immunoglobulin-like receptors (KIR) on the surface of NK cells and HLA-A, -B, an -C molecules on the surface of leukemic cells[10–12]. The KIR gene locus is characterized by high complexity, as it shows a high genetic variation in regard of receptor functions (activation or inhibition of target cell killing), gene content, copy number variation, and sequence polymorphism[13]. KIR expression profiles are further classified into two distinct haplotypes, A and B, based on the presence or absence of certain KIR genes[14;15]. The A-haplotype is defined by the presence of the two inhibitory KIRs KIR2DL1 and KIR2DL3, and KIR2DS4 as sole activating KIR. The B-haplotype is characterized by the presence of a variety of different combinations of KIR genes, however, the presence of at least one of the KIRs KIR2DL5, KIR2DS1/2/3/5, or KIR3DS1 is mandatory.

Possibly, the most important KIR-ligand interaction is that between HLA-C antigens and their corresponding KIRs, in which both, inhibitory and activating, receptors are involved [16]. However, HLA-C/KIR interactions appear to be even more complex, given that HLA-C antigens are divided into two ligand groups based on specific amino acid residues [17]: Antigens in the C1 group bear an asparagine residue on position 80 and interact with inhibitory KIR2DL2 and KIR2DL3 as well as activating KIR2DS2, whereas C2 group ligands carry a lysine residue at position 80 and interact with inhibitory KIR2DL1 and activating KIR2DS1([18;19]. Activating signals and missing inhibition, both, lead to lysis of target cells[20;21]. Lack of interaction between a KIR and its respective HLA-C ligand in conjunction with activating signals may induce lysis of the target cell, a mechanism which is known as “missing self-recognition”[22]. In fact, absence of one or more ligands for donor inhibitory KIRs in patients with acute myeloid leukemia (AML) was shown to have a positive effect on HSCT outcome in haploidentical transplantations[20;23]. Whereas this model is still controversially discussed in the context of matched HSCT[24–26], it has been shown that in matched unrelated transplantation the presence or absence of C1 and/or C2 ligands significantly affects outcome: patients who expressed at least one C1 ligand showed superior overall survival and lower TRM rates than patients who were homozygous for the C2 ligand[27].

In this multicentre study, we evaluated the C-ligand impact on unrelated HSCT outcome in a large cohort of 1446 KIR & 5-loci-high resolution HLA-typed HSCT transplant pairs and thereby confirmed the C1-ligand negative patient cohort to be at higher risk than C1-positive patients. However, the incidence of patient C2 homozygosity is relatively low (in our cohort 14%). Transplantation with KIR B-haplotype positive donors has already been shown to increase HSCT success [28–31]. KIR haplotype analyses that were performed in our lab confirm the beneficial effect of donor KIR B-haplotype on transplantation outcome (Abstract published in Biology of blood and marrow transplantation in 2015[32]). NK cells and their activating KIR molecules have also been shown to play a beneficial role in the immune response against various infectious agents[33–35] as well as in the killing of T-cell blasts[36] and the prevention of leukaemia relapse[37]. KIR-Haplotype organisation, however, unvailed highly various phenotypes, especially among individuals with KIR B-Haplotypes. In order to analyse the relative contribution of individual B-Haplotype defining KIRs on HSCT outcome, sub-analyses on the level of single KIR genes are required. In this study, we aimed to examine if C1-ligand negative patients, who were found to be at risk post transplantation, could benefit from transplantations carried out from donors with various activating KIRs.

## Patients and Methods

### Study cohort

The study population included a total of 1446 patients transplanted for malignant disorders with T-cell repleted grafts from unrelated donors between 2000 and 2009. Benign diseases of the hematopoietic system as well as inborn genetic diseases were excluded. Patients with AML (acute myeloid leukemia), ALL (acute lymphoblastic leukemia), AL (unclassified acute leukemia), CML (chronic myeloid leukemia), CLL (chronic lymphoblastic leukemia), MDS (myelodysplastic syndrome), and MM (multiple myeloma) were eligible. Disease stages were adopted from a previous report published by the European Society for Blood and Marrow Transplantation (EBMT) study group[38]: Early disease stage included AML, AL, and ALL transplanted in first complete remission, CML in first chronic phase, and NHL, MM, and MDS transplanted either untreated or in first complete remission. Intermediate disease stage was defined as AML, ALL, or NHL in second complete remission or first relapse, AL transplanted in second complete remission, lymphoma and MM in second complete remission, partial remission or stable disease, CML at all other stages than first chronic phase or blast crisis, and MDS in second complete remission or in partial remission. Advanced stage was asigned to AML, AL, ALL, and MDS in all other disease phases, CML in blast crisis, and lymphoma and MM in all other disease stages than early or intermediate.

Graft source was either peripheral blood stem cells or bone marrow. Only first allogeneic transplantations were included in the analysis. Transplant pairs were either 10/10 matched or single HLA-A, -B, or -C mismatched. All patients received myeloablative or reduced intensity conditioning. Myeloablative conditioning regimen was defined according to the EBMT recommendations as treatment with total body irradiation equal or above 10 Gy and/or cyclophosphamide equal or greater than 120 mg/m^2^, and/or busulfan equal or greater than 16 mg/kg[39;40]. Treatment with less intense regimen was classified as reduced intensity conditioning. All patients received T-cell replete grafts. Written recipient and donor consents for HLA typing and for the analysis of clinical data were obtained. Clinical data was provided by the German Registry for Stem Cell Transplantation (DRST) in pseudonymised form. This data was collected by the transplant centers at day 100 post transplantation and yearly thereafter, according to the EBMT guidelines. The study was approved by the ethical review board of the University of Ulm (project number 263/09). Overall survival (OS) was defined as time to death from any cause and was censored at the time of last follow-up. Disease-free survival (DFS) was defined as time to relapse of primary malignancy, or death from any cause, and was also censored at the time of last follow-up. Relapse incidence (RI) was defined as occurrence of relapse of hematologic malignancy at any given time point. Treatment-related mortality (TRM) was defined as mortality in complete disease remission.

### HLA-Typing in patients and donors

Donor-recipient pairs from unrelated donor searches completed before May 2005 were low resolution typed for HLA-A and -B, and high resolution typed for HLA-DRB1 and -DQB1. Stored DNA material of these individuals was retrospectively high resolution typed for HLA-A, -B, and -C. For unrelated donor searches which were initiated after 05/2005 5-loci high resolution HLA-typing was carried out upfront.

High resolution HLA-typing was performed by sequence based typing (SBT) using a CE-certified in-house Kit for HLA-class I (H-Seq-ABC, DRK-BSD Baden- Württemberg–Hessen gGmbH, Frankfurt am Main, Germany) and by in-house SBT (H-Seq-DR, DRK-BSD Baden- Württemberg–Hessen gGmbH, Frankfurt am Main, Germany and H-Seq-DQB, DRK-BSD Baden- Württemberg–Hessen gGmbH, Frankfurt am Main, Germany) or Luminex® (Life Technologies, Carlsbad, CA, USA) PCR-sequence specific oligonucleotide probe (PCR-SSO) method for HLA-class II. Exons 2 and 3 were sequenced for HLA-class I typing, and exon 2 was analysed for HLA-class II testing. Additional typing for relevant non-expressed alleles was performed according to the National Marrow Donor Program confirmatory typing requirements[41].

### KIR-Typing in patients and donors

KIR-typing was performed using the commercially available “KIR Genotyping SSP Kit” from Thermo Fisher Scientific (Waltham, MA, USA). The Kit uses sequence specific primer PCR to assign presence or absence, respectively, of a given KIR gene. The method allows the identification of the following KIR-genes: KIR2DL1/2/3/4/5A/5B, KIR2DS1/2/3/4/5, KIR3DL1/2/3, KIR3DS1, KIR2DP1, and KIR3DP1.

### Assessment of missing KIR ligand status

KIR ligand status of patients was obtained from high resolution HLA-typing. HLA-C molecules were grouped according to the presence of epitopes recognized by KIRs: HLA-C antigens with an asparagine residue at amino acid sequence position 80 possess the C1 epitope, which acts as ligand for the inhibitory KIR2DL2 and KIR2DL3 as well as for the activating KIR2DS2. Accordingly, HLA-C antigens with a lysine residue at this position possess the C2 epitope, which acts as ligand for the inhibitory KIR2DL1 as well as for the activating KIR2DS1.

The absence of C1 or C2 in patients who were transplanted with a KIR2DL2/3 or KIR2DL1 positive donor, respectively, was considered as “missing ligand”. Control patient groups carried at least one ligand for any donor KIR2DL1 or KIR2DL2/3, respectively.

### Statistical analysis

All statistical analyses were performed using the open source program for statistical computing “R”, version 3.0.2. Cumulative estimates of overall survival (OS) and disease free survival (DFS) were obtained using Kaplan-Meier analysis. Extended Cox regression models allowing for time-dependent covariates were used to conduct multivariate analyses of OS and DFS. Univariate analyses of relapse incidence (RI) and treatment related mortality (TRM) were performed using competing risk analysis. Competing risk regression models for stratified data were used for multivariate analysis of RI and TRM. The categorical variable diagnosis showed different baselines of hazard for the respective patient sub-groups. Hence, for both multivariate models stratification for diagnosis was accomplished rather than integrating diagnosis as a covariate[42;43]. Centre effects were adjusted using a gamma frailty term (fitted with the R-package “survival”)[44;45]. A stepwise backward exclusion procedure was used for model selection, where all variables have been included in the first model and then successively reduced (at each step the least significant) until the loss of information became significant (analysis of missing ligand model p≤0.05; KIR analyses p≤0.01)[43]. In order to address the problem of multiple testing and potentially associated type 1 errors we set the significance level for our KIR analyses to 0.01. Covariates were assessed according to previously published recommendations of the EBMT study group[42;43;46], including patient age, disease stage, graft manipulation, conditioning regimen, graft source, donor source (national vs. international), year of transplantation, time between diagnosis and transplantation, and donor-recipient gender combination. For the clinical covariates analysed, the data was complete. All models were checked for interactions and proportional hazards assumption. No significant interactions between covariates were found and violations of proportional hazards assumption were adjusted by using time dependent covariates.

## Results

### Patient characteristics

General patient characteristics are shown in Table 1. The cohort included 1446 transplant pairs. Median patient age was 52 years. Most patients were in early (38.5%) and intermediate (35.8%) disease state. Stem cell source was peripheral blood stem cells in 93.7% cases. Almost two thirds (60.2%) of the patients received myeloablative conditioning. An unfavourable donor-patient sex mismatch (female donor for a male patient) was found in 11.2% of transplant pairs. Most patients received a graft from a 10/10 matched HLA compatible donor (70.7%), whereas 29.3% were transplanted with a 9/10 matched donor. Median post-transplantation follow-up time was 40 months and maximum follow-up time was 9.5 years.

**Table 1:** General patient characteristics.

	n	%		n	%		n	%
Age			Disease stage			Patient and donor sex combination		
18–40	359	24.8%	Early	556	38.5%	Donor female, patient male	162	11.2%
41–60	1071	74.1%	Intermediate	518	35.8%	Others	1284	88.8%
61–76	16	1.1%	Advanced	372	25.7%			
HLA-compatibility			Graft source			Conditioning		
10/10	1022	70.7%	Bone Marrow	91	6.3%	Myeloablative	870	39.8%
9/10	424	29.3%	Peripheral Blood stem cells	1355	93.7%	Reduced intensity	576	60.2%
Year of transplantation			Diagnosis myeloid			Diagnosis lymphoblastic		
2000–2005	377	26.1%	AML	447	30.9%	ALL	189	13.1%
2006–2009	1069	73.9%	CML	65	4.5%	CLL	82	5.7%
			MDS	271	18.7%	MM	131	9.1%
						NHL	176	12.2%
			Diagnosis other					
			AL	85	5.9%			

Table 2 lists the distribution of activating KIR2DS1/2/3/5 and KIR3DS1 among donors subject to subgroups of patients with myeloid or lymphoid malignancies. Between 54 and 60% of donors were positive for KIR2DS1. KIR2DS2 was even more prevalent with up to 75% being carriers. KIR2DS3 and KIR2DS5 were found in 40–50% of donors and KIR3DS1 was present in more than 50% of donors.

**Table 2:** Distribution of activating KIR2DS1/2/3/5 and KIR3DS1 among donors in combination with patient C1-ligand status in the study cohort.

	Myeloid malignancy		Lymphoid malignancy	
Donor	Patient C1-positive	Patient C1-negative	Patient C1-positive	Patient C1-negative
	n = 666 (85.1%)	n = 117 (14.9%)	n = 501 (86.7%)	n = 77 (13.3%)
KIR2DS1 positive	258 (38.7%)	45 (38.5%)	200 (39.9%)	28 (36.4%)
KIR2DS1 negative	408 (61.3%)	72 (61.5%)	301 (60.1%)	49 (63.6%)
KIR2DS2 positive	314 (47.1%)	63 (53.8%)	246 (49.1%)	35 (45.4%)
KIR2DS2 negative	352 (52.9%)	54 (45.2%)	255 (50.9%)	42 (54.5%)
KIR2DS3 positive	161 (24.2%)	41 (35.0%)	130 (25.9%)	19 (24.7%)
KIR2DS3 negative	505 (75.8%)	76 (65.0%)	371 (74.1%)	58 (75.3%)
KIR2DS5 positive	202 (30.3%)	34 (29.1%)	152 (30.3%)	21 (27.3%)
KIR2DS5 negative	464 (69.7%)	83 (70.9%)	349 (69.7%)	56 (72.7%)
KIR3DS1 positive	229 (34.4%)	45 (38.5%)	186 (37.1%)	30 (39.0%)
KIR3DS1 negative	437 (65.6%)	72 (61.5%)	315 (62.9%)	47 (61.0%)

### C1-negative patients show significantly inferior HSCT outcome

Distribution of KIR2DL1/2/3 and HLA-C-ligands among patients and donors is shown in Table 3. All individuals in the cohort were either positive for KIR2DL2 or KIR2DL3, or both (approximately 40%). The corresponding C1 ligand for these KIRs was carried in 86% of the individuals. Of these, 40% were C1 homozygous. KIR2DL1 was detected in 96% of patients and donors. The C2 ligand was detected in 60% of the patients. Homozygosity for this ligand was relatively rare with a prevalence of 13.8%.

**Table 3:** Distribution of KIR2DL1, KIR2DL2 and KIR2DL3 as well as C1/C2 genotype in the study cohort.

	KIR2DL1	KIR2DL2	KIR2DL3	KIR2DL2/3	Both ligands present	Missing C2 ligand	Missing C1 ligand
Patients	1393	762	1302	618	663	583	200
	(96.3%)	(52.7%)	(90.0%)	(42.7%)	(45.9%)	(40.3%)	(13.8%)
Donors	1388	720	1307	581	673	578	195
	(96.0%)	(49.8%)	(90.4%)	(40.2%)	(46.5%)	(40.0%)	(13.5%)

**Fig 1:**
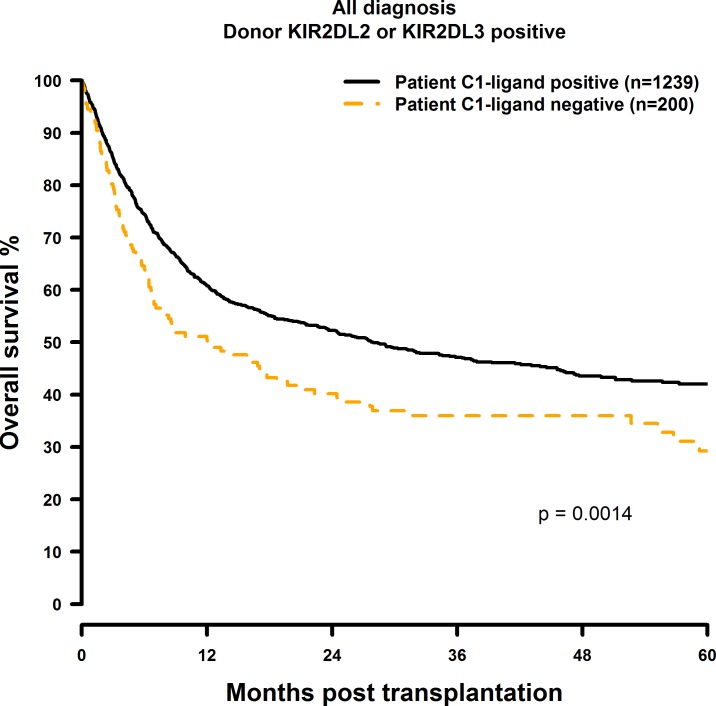
Univariate OS analysis in C1-negative patients (missing C1 ligand) vs. C1-positive patients who were transplanted with KIR2DL2 or KIR2DL3 positive donors. Dashed orange line: C1-negative patients (n = 200). Solid black line: C1-positive patients (n = 1239). p = 0.0014.

**Fig 2:**
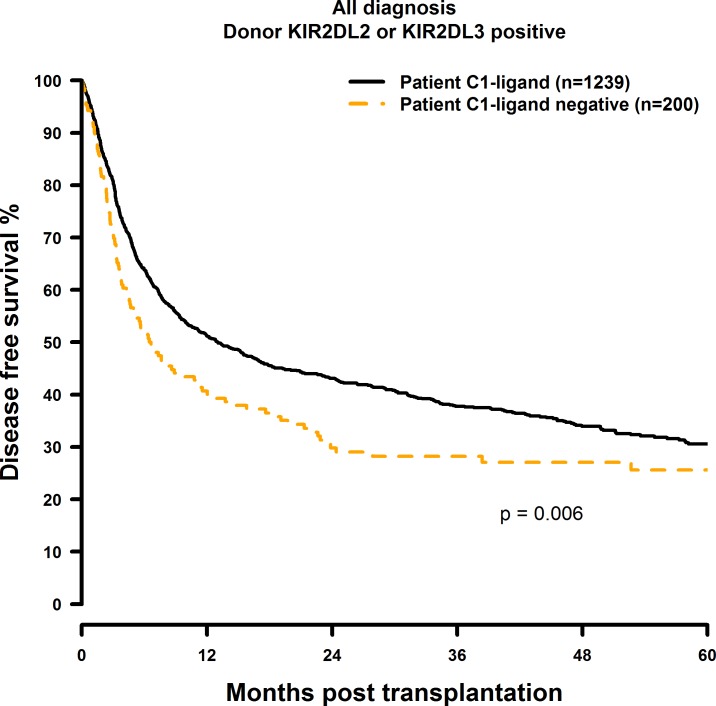
Univariate DFS analysis in C1-negative patients (missing C1 ligand) vs. C1-positive patients who were transplanted with KIR2DL2 or KIR2DL3 positive donors. Dashed orange line: C1-negative patients (n = 200). Solid black line: C1-positive patients (n = 1239). p = 0.006.

**Fig 3:**
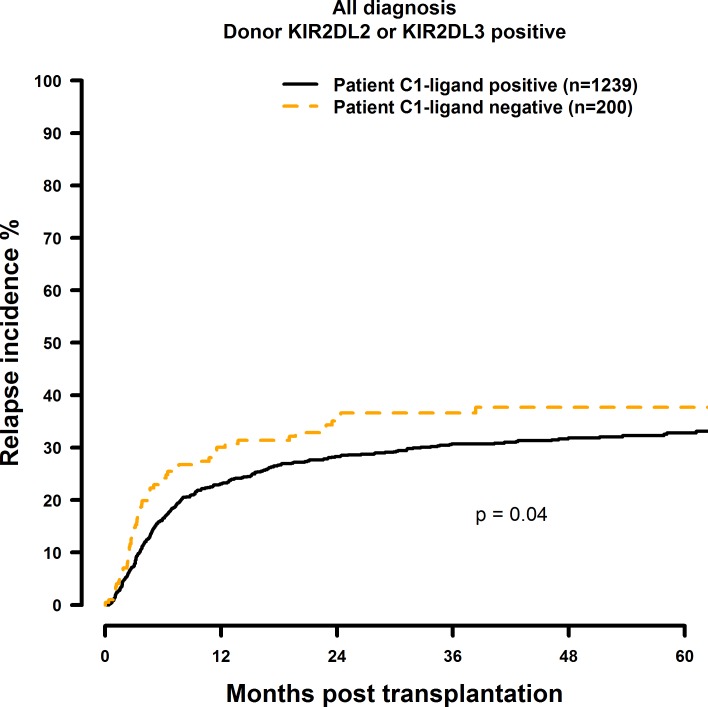
Univariate RI analysis in C1-negative patients (missing C1 ligand) vs. C1-positive patients who were transplanted with KIR2DL2 or KIR2DL3 positive donors. Dashed orange line: C1-negative patients (n = 200). Solid black line: C1-positive patients (n = 1239). p = 0.04.

**Fig 4:**
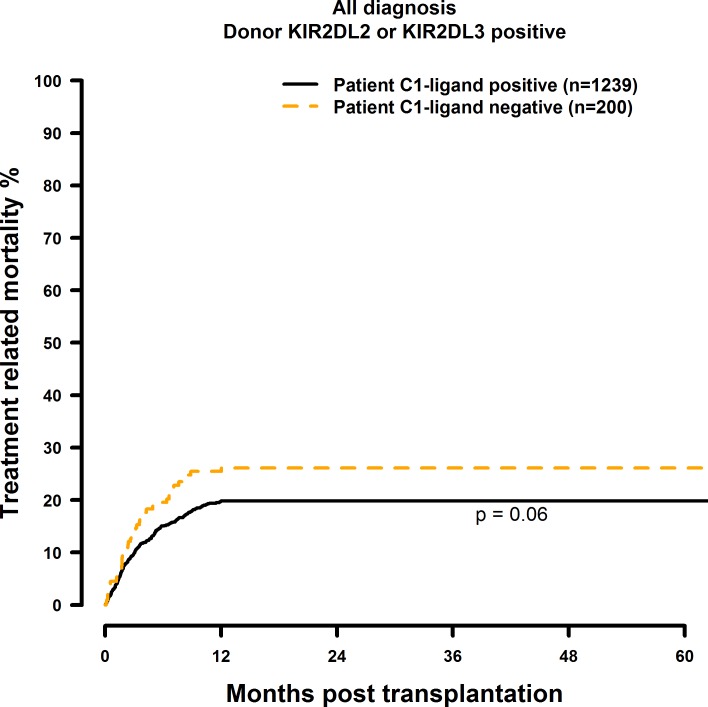
Univariate TRM analysis in C1-negative patients (missing C1 ligand) vs. C1-positive patients who were transplanted with KIR2DL2 or KIR2DL3 positive donors. Dashed orange line: C1-negative patients (n = 200). Solid black line: C1-positive patients (n = 1239). p = 0.06.

Patients who did not possess the C1 ligand (i.e. C2 homozygous) showed significantly inferior OS and DFS after 1, 3 and 5 years post transplantation (Fig 1 and Fig 2) when compared to the C1 positive (C1 homozygous or C1C2 heterozygous) control group (OS: 60.7% vs. 49.7%; 47.0% vs. 36.0%; 41.6% vs. 29.2%, p = 0.0014. DFS: 51.1% vs. 40.0%; 37.6% vs. 27.1%; 30.3% vs. 26.6%, p = 0.006; Table 4). C2 Homozygosity also was associated with increased RI and TRM rates as shown in Fig 3 and Fig 4. Multivariate analysis confirmed these results, since OS (HR = 1.41, CI = 1.14–1.74, p = 0.0012), DFS (HR = 1.27, CI = 1.05–1.53, p = 0.015), RI (HR = 1.33, CI = 1.01–1.75, p = 0.04) and TRM (HR = 1.41, CI = 1.01–1.96, p = 0.04) all were negatively affected when the patients did not possess the C1 ligand (Table 4).

**Table 4:** Multivariate analysis of OS, DFS, RI, and TRM in C1-negative patients vs. patients with at least one C1 ligand.

Endpoint	Cases	HR	95% CI	p
OS	200	1.41	1.14–1.74	0.0012
DFS	200	1.27	1.05–1.53	0.015
RI	200	1.33	1.01–1.75	0.04
TRM	200	1.41	1.01–1.96	0.04

A significant impact of the missing ligand model described in previous studies was solely found for myeloid malignancies. The four endpoints were therefore re-analysed in subgroups of patients with myeloid and lymphoblastic diseases, respectively.

The protective effect of C1 could be observed only in the myeloid group (Table 5), where absence of C1 was associated with reduced OS (myeloid: HR = 1.51, CI = 1.15–1.99, p = 0.003; lymphoblastic: HR = 1.26, CI = 0.91–1.75, p = 0.16), reduced DFS (myeloid: HR = 1.31, CI = 1.01–1.70, p = 0.04; lymphoblastic: HR = 1.21, CI = 0.90–1.61, p = 0.21), and increased RI (myeloid: HR = 1.31, CI = 1.01–1.70, p = 0.04; lymphoblastic: HR = 1.21, CI = 0.90–1.61, p = 0.21). There were no differences observed for TRM between the myeloid and lymphoblastic subgroups (myeloid: HR = 1.34, CI = 0.85–2.11, p = 0.20; lymphoblastic: HR = 1.27, CI = 0.75–2.16, p = 0.38).

**Table 5:** Multivariate analysis of OS, DFS, RI, and TRM in C1-negative patients with myeloid or lymphoid diseases vs. control group patients with at least one C1 ligand.

Endpoint	Cases	HR	95% CI	p
OS myeloid	117	1.51	1.15–1.99	0.003
OS lymphoid	83	1.26	0.91–1.75	0.16
DFS myeloid	117	1.31	1.01–1.70	0.04
DFS lymphoid	83	1.21	0.90–1.61	0.21
RI myeloid	117	1.31	1.01–1.70	0.04
RI lymphoid	83	1.21	0.90–1.61	0.21
TRM myeloid	117	1.34	0.85–2.11	0.20
TRM lymphoid	83	1.27	0.75–2.16	0.38

C1-negative patients with myeloid malignancies were thus identified as being at risk and therefore this patient group is referred as such in subsequent analyses.

### Missing C2-ligand has no effect on HSCT outcome

**Fig 5:**
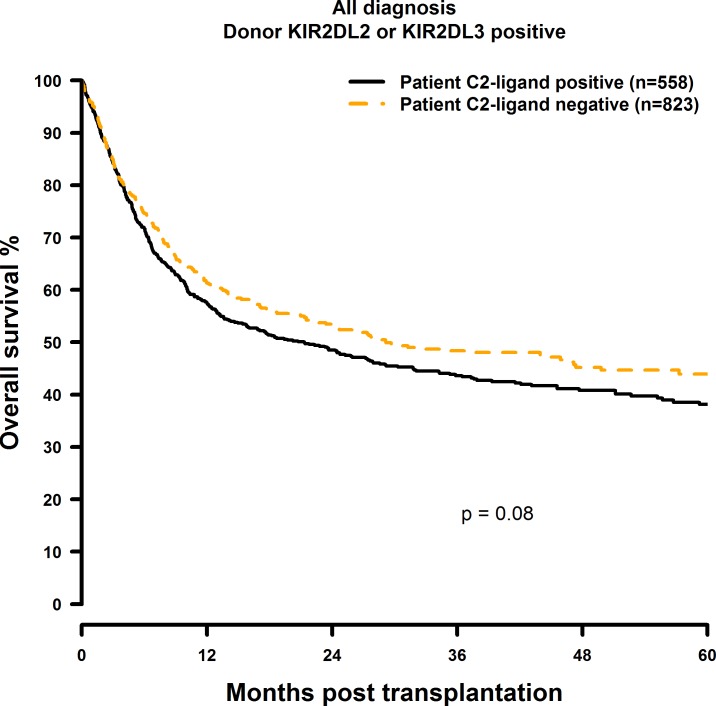
Univariate OS analysis in C2-negative (missing C2 ligand) vs. C2-positive patients who were transplanted with KIR2DL1-positive donors. Dashed orange line: C2-negative patients (n = 823). Solid black line: C2-positive patients (n = 558). p = 0.08.

**Fig 6:**
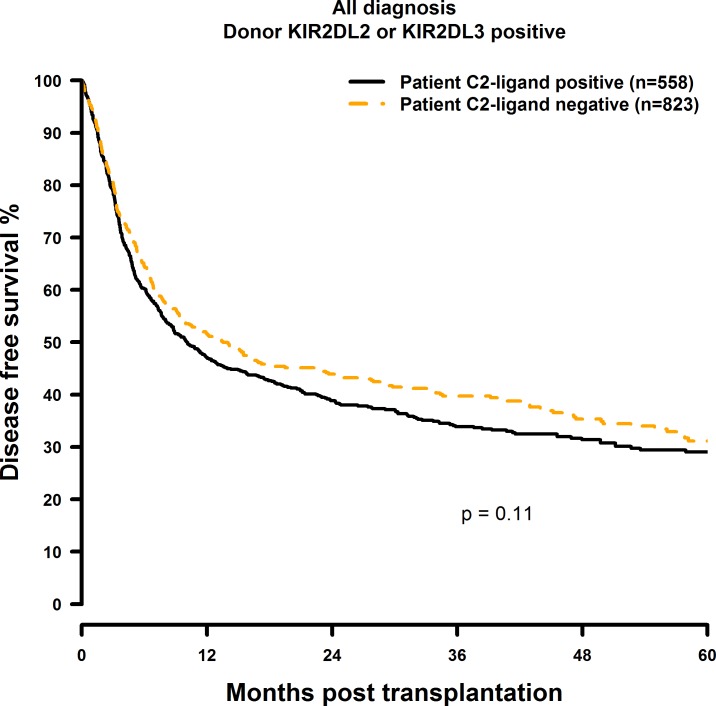
Univariate DFS analysis in C2-negative (missing C2 ligand) vs. C2-positive patients who were transplanted with KIR2DL1-positive donors. Dashed orange line: C2-negative patients (n = 823). Solid black line: C2-positive patients (n = 558). p = 0.11.

**Fig 7:**
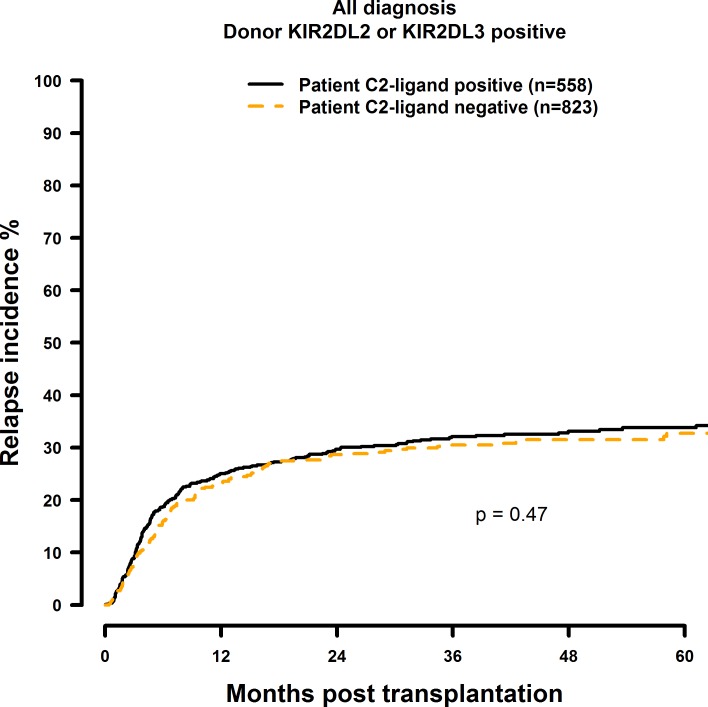
Univariate RI analysis in C2-negative (missing C2 ligand) vs. C2-positive patients who were transplanted with KIR2DL1-positive donors. Dashed orange line: C2-negative patients (n = 823). Solid black line: C2-positive patients (n = 558). p = 0.47.

**Fig 8:**
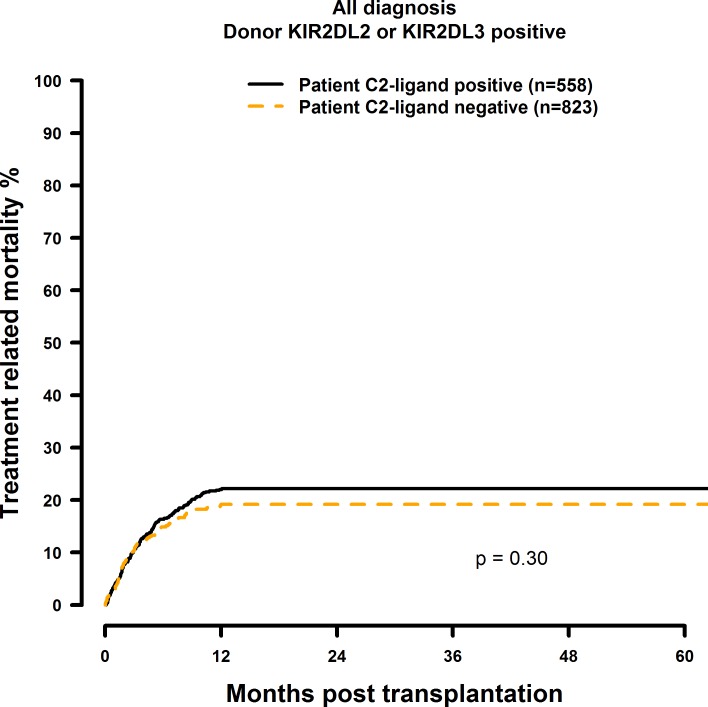
Univariate TRM analysis in C2-negative (missing C2 ligand) vs. C2-positive patients who were transplanted with KIR2DL1-positive donors. Dashed orange line: C2-negative patients (n = 823). Solid black line: C2-positive patients (n = 558). p = 0.30.

Missing C2-ligand does not affect HSCT outcome as shown by Kaplan-Meier analysis (Figs 5–8) and multivariate analysis (OS: HR = 0.91, CI = 0.77–1.03, p = 0.22; DFS: HR = 0.94, CI = 0.81–1.09, p = 0.41; RI: HR = 0.97, CI = 0.79–1.19, p = 0.74; TRM: HR = 0.90, CI = 0.69–1.16, p = 0.42).

### Transplantation with KIR2DS2 positive donors significantly increases OS and DFS in the C1-negative risk group

Since C1-deficiency was shown to negatively affect HSCT outcome in patients with myeloid, but not lymphoblastic, malignancies, following analyses were conducted in the myeloid patient group, only.

As KIR2DS4 is present in almost all individuals and is expressed on both, A- and B-Haplotypes, this KIR gene was not taken into consideration for any analyses in our study. Additionally, OS and DFS analyses were conducted in subgroups of 10/10 and 9/10 matched transplants.

**Fig 9:**
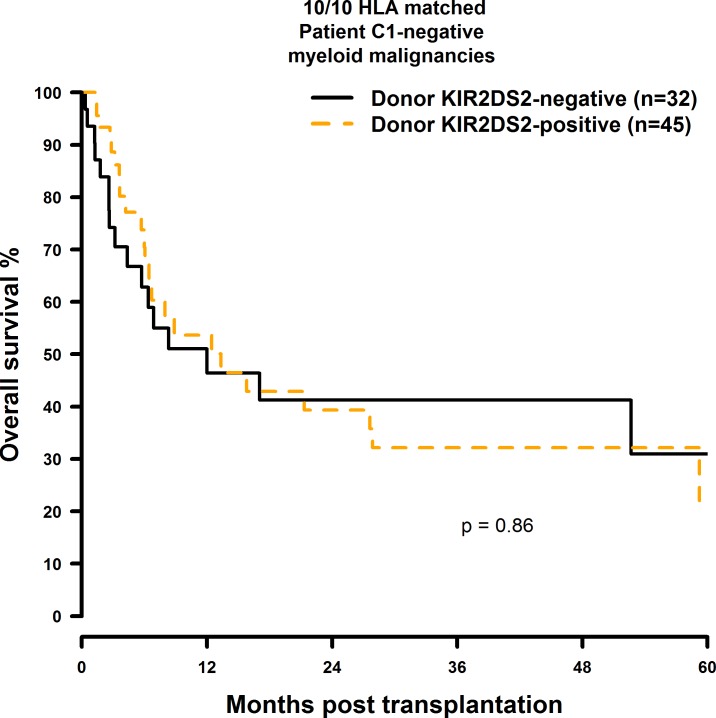
Univariate OS analysis in C1-negative patients with myeloid malignancies who were transplanted with KIR2DS2 positive donors vs. KIR2DS2 negative donors. Solid black line: donor KIR2DS2-negative. Dashed orange line: donor KIR2DS2-positive. 10/10 matched transplant pairs, donor KIR2DS2-negative (n = 32) vs. donor KIR2DS2-positive (n = 45), p = 0.86.

**Fig 10:**
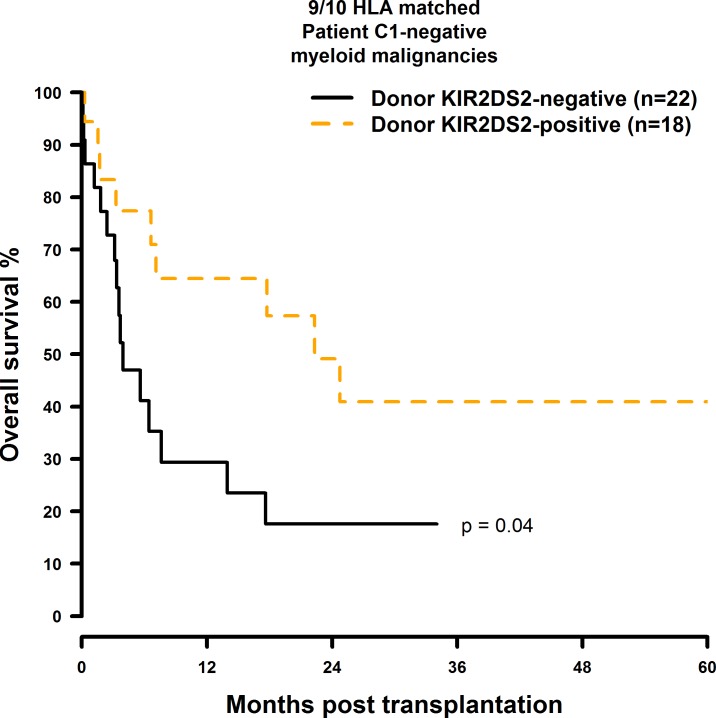
Univariate OS analysis in C1-negative patients with myeloid malignancies who were transplanted with KIR2DS2 positive donors vs. KIR2DS2 negative donors. Solid black line: donor KIR2DS2-negative. Dashed orange line: donor KIR2DS2-positive. 9/10 matched transplant pairs, donor KIR2DS2-negative (n = 22) vs. donor KIR2DS2-positive (n = 18), p = 0.04.

**Fig 11:**
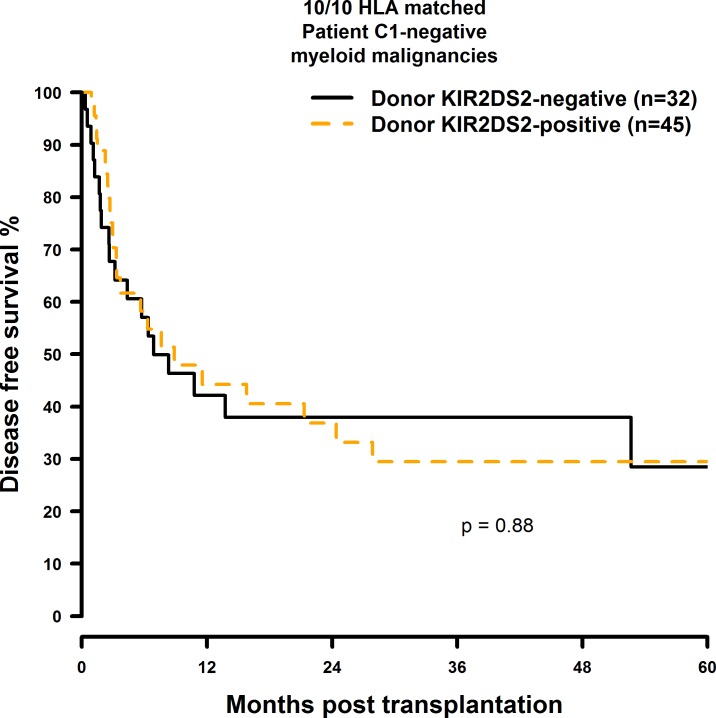
DFS analysis in C1-negative patients with myeloid malignancies who were transplanted with KIR2DS2 positive donors vs. KIR2DS2 negative donors. Solid black line: donor KIR2DS2-negative. Dashed orange line: donor KIR2DS2-positive. 10/10 matched transplant pairs, donor KIR2DS2-negative (n = 32) vs. donor KIR2DS2-positive (n = 45), p = 0.88.

**Fig 12:**
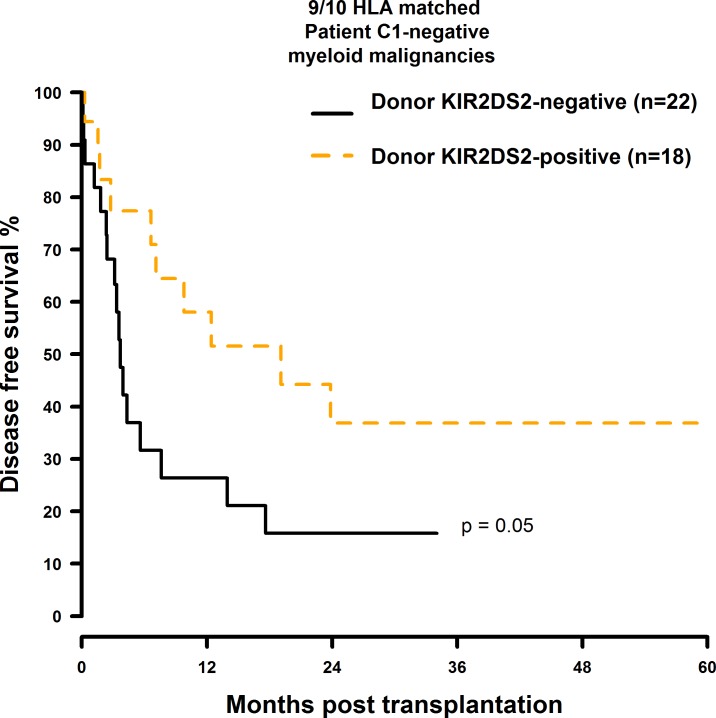
DFS analysis in C1-negative patients with myeloid malignancies who were transplanted with KIR2DS2 positive donors vs. KIR2DS2 negative donors. Solid black line: donor KIR2DS2-negative. Dashed orange line: donor KIR2DS2-positive. 9/10 matched transplant pairs, donor KIR2DS2-negative (n = 22) vs. donor KIR2DS2-positive (n = 18), p = 0.05.

OS and DFS were significantly improved when the donor was positive for KIR2DS2 and the transplant pair was mismatched at a single HLA-class I gene: The OS rates were twice as high as in the 9/10 matched patient group with a KIR2DS2 negative donor (10/10: 49.5% vs. 44.7%; 38.5% vs. 34.1%; 28.9% vs. 25.6%; 9/10: 57.3% vs. 23.5%; 40.9% vs. 17.6%; Fig 9 and Fig 10). Similarly, donor KIR2DS2 positivity resulted in higher DFS rates in the 9/10 matched patient group (10/10: 40.6% vs. 37.9%; 29.5% vs. 28.4%; 9/10: 51.6% vs. 21.1%; 24.6% vs. 15.8%; Fig 11 and Fig 12). Multivariate analysis confirmed these results (OS: 10/10: HR = 1.02, CI = 0.51–2.07, p = 0.95; 9/10: HR = 0.24, CI = 0.08–0.67, p = 0.007; DFS: 10/10: HR = 1.23, CI = 0.72–2.07, p = 0.44; 9/10: HR = 0.26, CI = 0.11–0.60, p = 0.002; Table 6 and 6B). Reduced intensity conditioning regimen was a significant predictor within our analysis (S1 Table) and it would have been interesting to investigate whether the influence of KIR2DS2 could be observed in both, reduced intensity and myeloablative conditioning, respectively. As individual disease stages (early, intermediate, and advanced) are important predictors for HSCT outcome as well, it would have also been interesting to investigate the effect of donor KIR2DS2 positivity in according to disease stage. Unfortunately the number of cases in our study did not allow these analyses.

**Table 6:** Multivariate analysis of OS in C1-negative patients transplanted with a KIR2DS1/2/3/5 or KIR3DS1 positive donor vs. C1-negative patients transplanted with a KIR2DS1/2/3/5 or KIR3DS1 negative donor.

	Cases	HR	95% CI	p
Donor KIR2DS1 negative 10/10	47	1	-	-
Donor KIR2DS1 positive 10/10	30	0.75	0.37–1.52	0.42
Donor KIR2DS1 negative 9/10	25	1	-	-
Donor KIR2DS1 positive 09/10	15	1.88	0.74–4.76	0.18
Donor KIR2DS2 negative 10/10	32	1	-	-
Donor KIR2DS2 positive 10/10	45	1.04	0.51–2.12	0.91
Donor KIR2DS2 negative 9/10	22	1	-	-
Donor KIR2DS2 positive 9/10	18	0.24	0.08–0.67	0.007
Donor KIR2DS3 negative 10/10	49	1	-	-
Donor KIR2DS3 positive 10/10	28	1.38	0.68–2.79	0.37
Donor KIR2DS3 negative 9/10	27	1	-	-
Donor KIR2DS3 positive 9/10	13	0.85	0.31–2.37	0.76
Donor KIR2DS5 negative 10/10	56	1	-	-
Donor KIR2DS5 positive 10/10	21	0.55	0.25–1.24	0.15
Donor KIR2DS5 negative 9/10	27	1	-	-
Donor KIR2DS5 positive 9/10	13	2.19	0.78–6.12	0.13
Donor KIR3DS1 negative 10/10	46	1	-	-
Donor KIR3DS1 positive 10/10	31	0.96	0.47–1.97	0.92
Donor KIR3DS1 negative 9/10	26	1	-	-
Donor KIR3DS1 positive 9/10	14	1.74	0.66–4.56	0.26

Presence of other activating KIR KIR2DS1/3/5 or KIR3DS1 in the donor did not have any effect on OS or DFS (Tables 6 and 7).

**Table 7:** Multivariate analysis of DFS in C1-negative patients transplanted with a KIR2DS1/2/3/5 or KIR3DS1 positive donor vs. C1-negative patients transplanted with a KIR2DS1/2/3/5 or KIR3DS1 negative donor.

	Cases	HR	95% CI	p
Donor KIR2DS1 negative 10/10	47	1	-	-
Donor KIR2DS1 positive 10/10	30	0.65	0.38–1.11	0.11
Donor KIR2DS1 negative 9/10	25	1	-	-
Donor KIR2DS1 positive 09/10	15	1.31	0.66–2.59	0.44
Donor KIR2DS2 negative 10/10	32	1	-	-
Donor KIR2DS2 positive 10/10	45	1.23	0.72–2.07	0.44
Donor KIR2DS2 negative 9/10	22	1	-	-
Donor KIR2DS2 positive 9/10	18	0.26	0.11–0.60	0.002
Donor KIR2DS3 negative 10/10	49	1	-	-
Donor KIR2DS3 positive 10/10	28	1.27	0.75–2.13	0.37
Donor KIR2DS3 negative 9/10	27	1	-	-
Donor KIR2DS3 positive 9/10	13	0.67	0.33–1.37	0.27
Donor KIR2DS5 negative 10/10	56	1	-	-
Donor KIR2DS5 positive 10/10	21	1.14	0.64–2.02	0.66
Donor KIR2DS5 negative 9/10	27	1	-	-
Donor KIR2DS5 positive 9/10	13	1.36	0.65–2.88	0.41
Donor KIR3DS1 negative 10/10	46	1	-	-
Donor KIR3DS1 positive 10/10	31	0.84	0.49–1.45	0.54
Donor KIR3DS1 negative 9/10	26	1	-	-
Donor KIR3DS1 positive 9/10	14	1.24	0.62–2.51	0.54

### Transplantation with KIR2DS1 positive donors is associated with a significantly decreased RI in the C1-negative risk patient group

For RI and TRM analyses, case numbers were too small to conduct sub-analysis in 10/10 and 9/10 matched transplant pairs. Therefore the subsequent analyses of the impact of KIR2DS1/2/3/5 or KIR3DS1 positive grafts on RI and TRM within the risk patient group were conducted disregarding HLA matching degree (univariate analysis) or with stratification for HLA matching (multivariate analysis).

**Fig 13:**
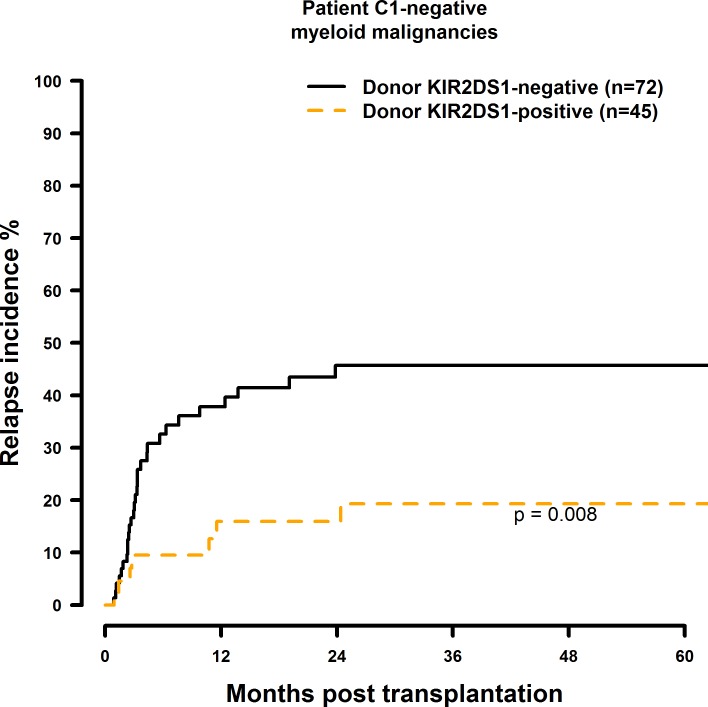
Univariate RI analysis in C1-negative patients with myeloid malignancies who were transplanted with KIR2DS1 positive vs. KIR2DS1 negative donors. Solid black line: donor KIR2DS1 negative. Dashed orange line: donor KIR2DS1 positive. Donor KIR2DS1-negative (n = 72) vs. donor KIR2DS1-positive (n = 45), p = 0.008.

**Fig 14:**
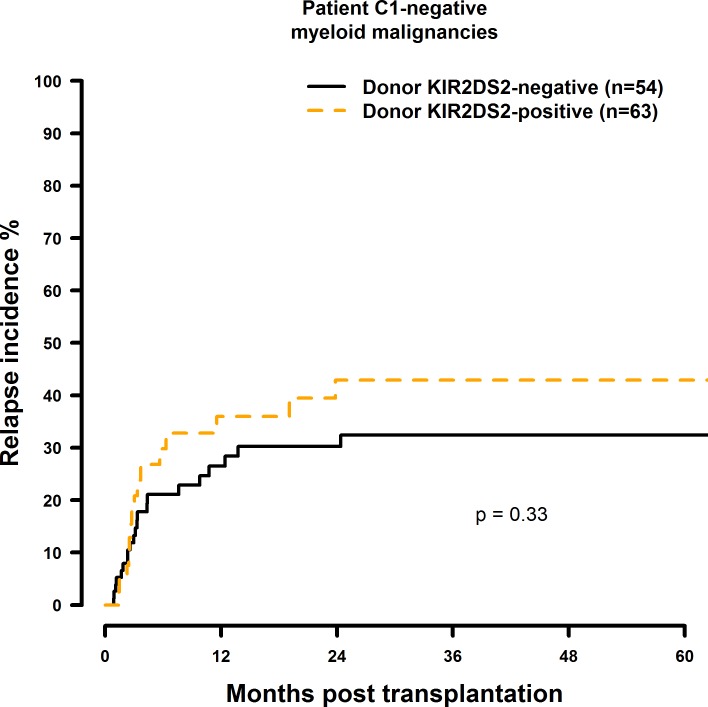
Univariate RI analysis in C1-negative patients with myeloid malignancies who were transplanted with KIR2DS2 positive vs. KIR2DS2 negative donors. Solid black line: donor KIR2DS2 negative. Dashed orange line: donor KIR2DS2 positive. Donor KIR2DS2-negative (n = 54) vs. donor KIR2DS2-positive (n = 63), p = 0.33.

**Fig 15:**
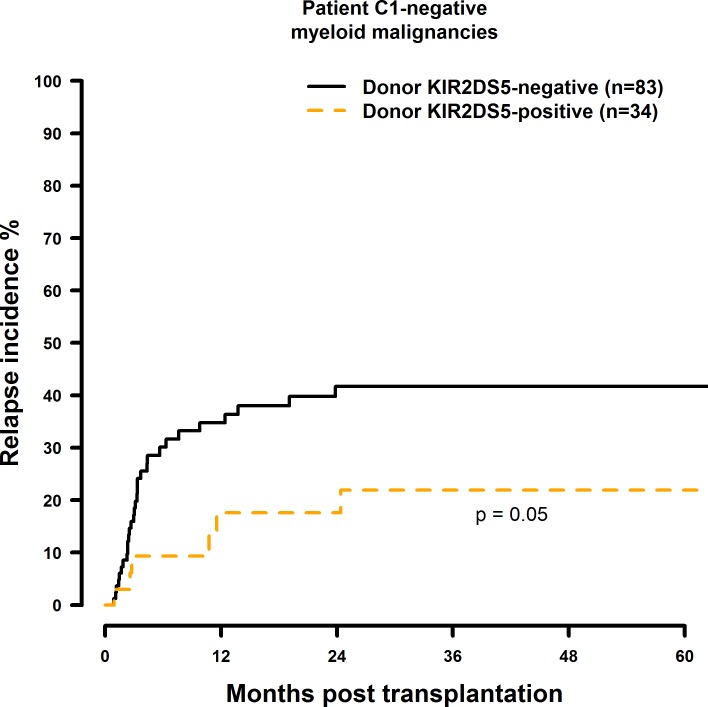
Univariate RI analysis in C1-negative patients with myeloid malignancies who were transplanted with KIR2DS5 positive vs. KIR2DS5 negative donors. Solid black line: donor KIR2DS5 negative. Dashed orange line: donor 5 positive. Donor KIR2DS5-negative (n = 83) vs. donor KIR2D5-positive (n = 34), p = 0.05.

**Fig 16:**
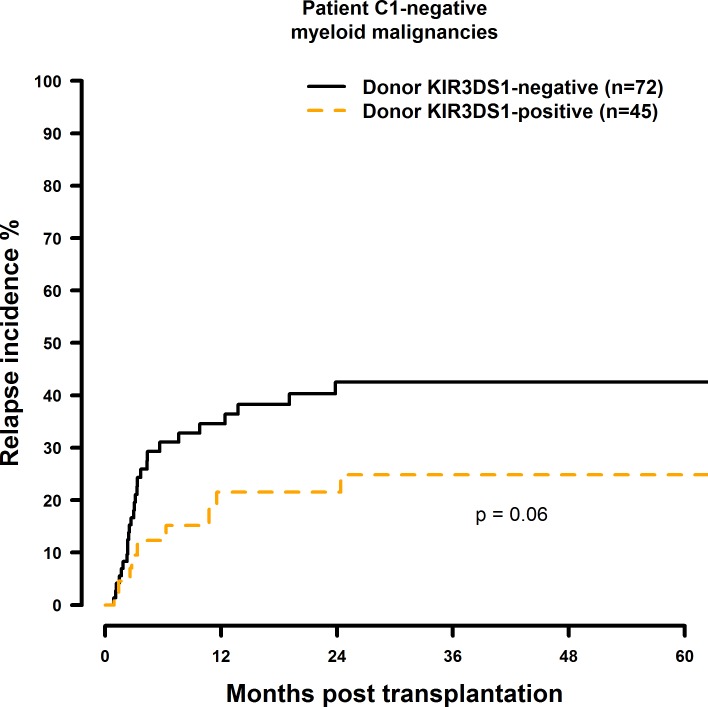
Univariate RI analysis in C1-negative patients with myeloid malignancies who were transplanted with KIR3DS1 positive vs. KIR3DS1 negative donors. Solid black line: donor KIR3DS1 negative. Dashed orange line: donor KIR3DS1 positive. Donor KIR3DS1-negative (n = 72) vs. donor KIR3DS1-positive (n = 45), p = 0.06.

Transplantation with KIR2DS1 positive donors significantly reduced RI in C1-negative patients (HR = 0.30, CI = 0.13–0.69, p = 0.005) (Fig 13, Table 8). Transplantation with a KIR2DS2-positive (HR = 1.87, CI = 0.86–4.08, p = 0.12), KIR2DS3-positive (HR = 1.60, CI = 0.83–3.10, p = 0.16), KIR2DS5-positive (HR = 0.37, CI = 0.15–0.92, p = 0.03), or KIR3DS1-positive (HR = 0.51, CI = 0.24–1.10, p = 0.09) donor had no significant effect on RI (Figs 14–16, Table 8).

**Table 8:** Multivariate analysis of RI in C1-negative patients transplanted with a KIR2DS1/2/3/5 or KIR3DS1 positive donor vs. C1-negative patients transplanted with a KIR2DS1/2/3/5/or KIR3DS1 negative donor.

	Cases	HR	95% CI	p
Donor KIR2DS1 negative	72	1	-	-
Donor KIR2DS1 positive	45	0.30	0.13–0.69	0.005
Donor KIR2DS2 negative	54	1	-	-
Donor KIR2DS2 positive	63	1.87	0.86–4.08	0.12
Donor KIR2DS3 negative	76	1	-	-
Donor KIR2DS3 positive	41	1.60	0.83–3.10	0.16
Donor KIR2DS5 negative	83	1	-	-
Donor KIR2DS5 positive	34	0.37	0.15–0.92	0.03
Donor KIR3DS1 negative	72	1	-	-
Donor KIR3DS1 positive	45	0.51	0.24–1.40	0.09

### Transplantation with KIR2DS1 positive donors is associated with a significantly increased TRM in the C1-negative risk patient group

**Fig 17:**
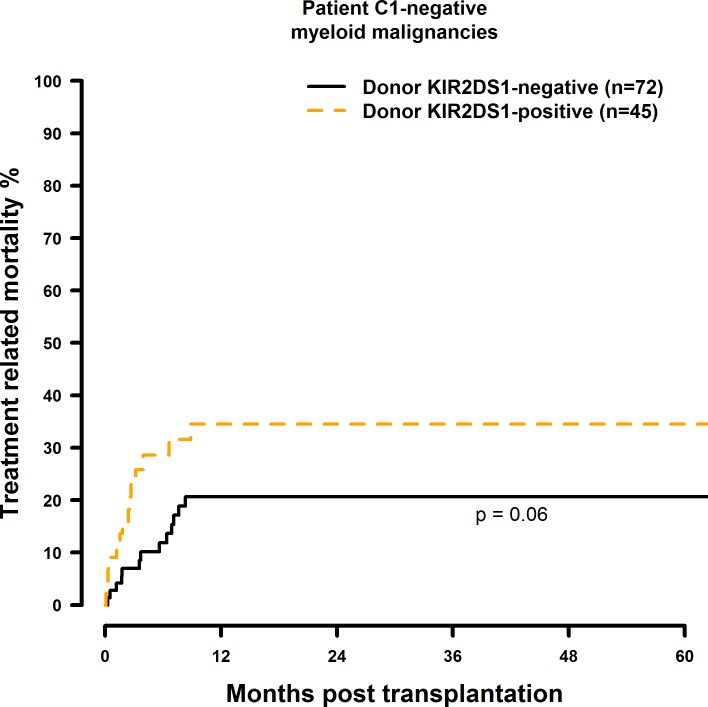
Univariate TRM analysis in C1-negative patients with myeloid malignancies who were transplanted with KIR2DS1 positive vs. KIR2DS1 negative donors. Solid black line: donor KIR2DS1 negative. Dashed orange line: donor KIR2DS1 positive. Donor KIR2DS1-negative (n = 72) vs. donor KIR2DS1-positive (n = 45), p = 0.06.

**Fig 18:**
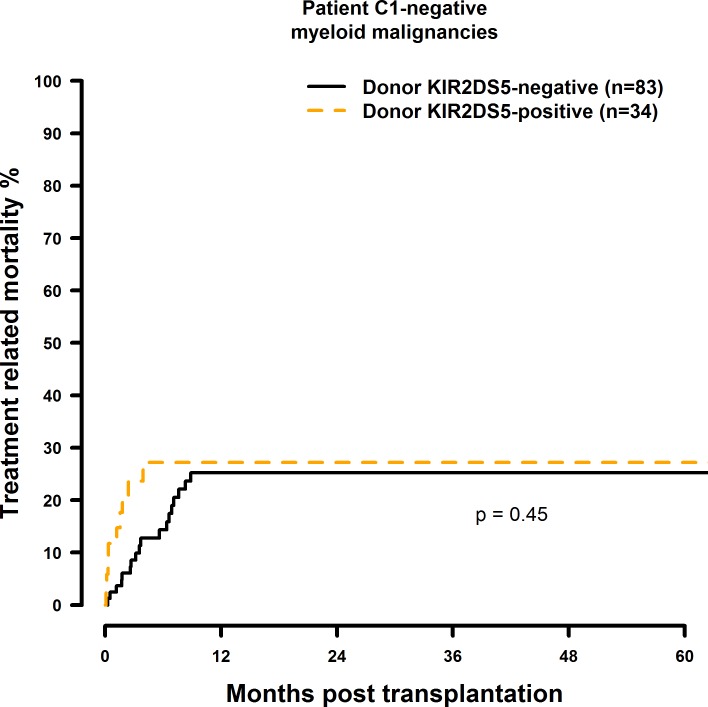
Univariate TRM analysis in C1-negative patients with myeloid malignancies who were transplanted with KIR2DS5 positive vs. KIR2DS5 negative donors. Solid black line: donor KIR2DS5 negative. Dashed orange line: donor KIR2DS5 positive. Donor KIR2DS5-negative (n = 83) vs. donor KIR2D5-positive (n = 34), p = 0.45.

**Fig 19:**
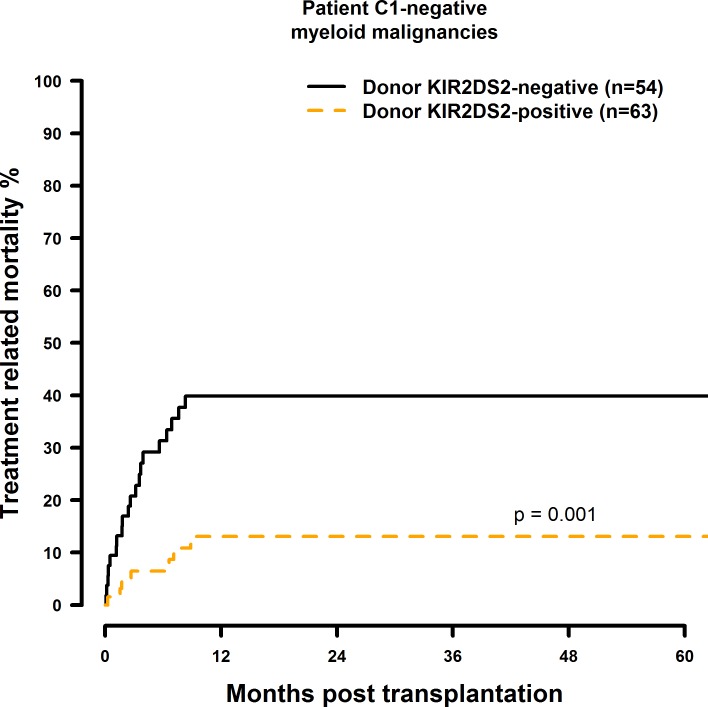
Univariate TRM analysis in C1-negative patients with myeloid malignancies who were transplanted with KIR2DS2 positive vs. KIR2DS2 negative donors. Solid black line: donor KIR2DS2 negative. Dashed orange line: donor KIR2DS2 positive. Donor KIR2DS2-negative (n = 54) vs. donor KIR2DS2-positive (n = 63), p = 0.001.

**Fig 20:**
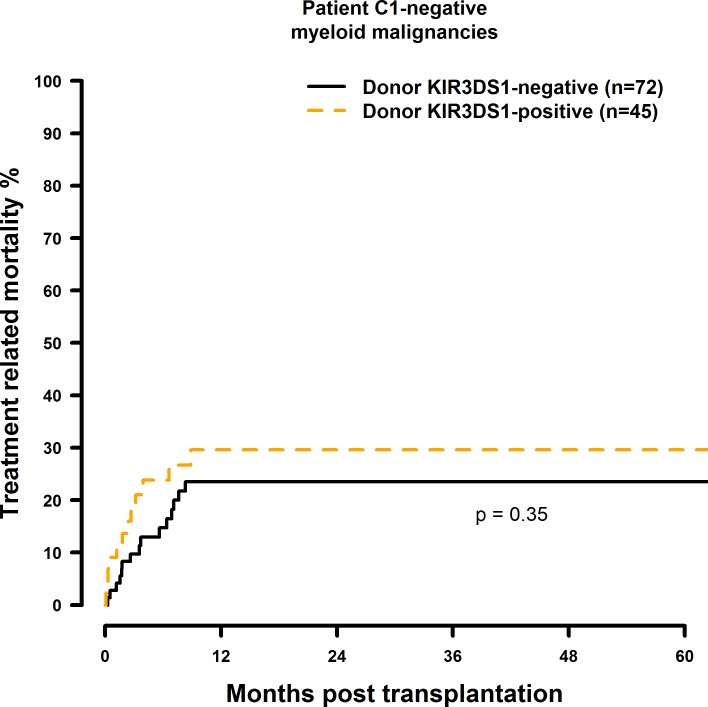
Univariate TRM analysis in C1-negative patients with myeloid malignancies who were transplanted with KIR3DS1 positive vs. KIR3DS1 negative donors. Solid black line: donor KIR3DS1 negative. Dashed orange line: donor KIR3DS1 positive. Donor KIR3DS1-negative (n = 72) vs. donor KIR3DS1-positive (n = 45), p = 0.35.

The results of the multivariate TRM analyses are summarised in Table 9. In the presence of a single HLA class I mismatch, TRM was increased in C1 negative patients who were transplanted with a KIR2DS1 (HR = 2.61, CI = 1.26–5.41, p = 0.001; Fig 17) positive donor. Transplantation with a KIR2DS5 positive donor had no effect on TRM (HR = 0.81, CI = 0.36–1.86, p = 0.63, Fig 18) and a potential positive effect of a KIR2DS2 positive graft (HR = 0.31, CI = 0.11–0.86, p = 0.03, Fig 19) as well as the effects observed on TRM by selecting a KIR2DS5 (HR = 3.33, CI = 1.20–9.29, p = 0.02), or KIR3DS1 (HR = 2.18, CI = 0.99–4.78, p = 0.05, Fig 20) positive donor, respectively, did not reach the level of statistical significance (0.01).

**Table 9:** Multivariate analysis of TRM in C1-negative patients transplanted with a KIR2DS1/2/3/5 or KIR3DS1 positive donor vs. C1-negative patients transplanted with a KIR2DS1/2/3/5/or KIR3DS1 negative donor.

	Cases	HR	95% CI	p
Donor KIR2DS1 negative	72	1	-	-
Donor KIR2DS1 positive	45	2.61	1.26–5.41	0.001
Donor KIR2DS2 negative	54	1	-	-
Donor KIR2DS2 positive	63	0.31	0.11–0.86	0.03
Donor KIR2DS3 negative	76	1	-	-
Donor KIR2DS3 positive	41	0.81	0.36–1.86	0.63
Donor KIR2DS5 negative	83	1	-	-
Donor KIR2DS5 positive	34	3.33	1.20–9.29	0.02
Donor KIR3DS1 negative	72	1	-	-
Donor KIR3DS1 positive	45	2.18	0.99–4.78	0.05

### The KIR Better/Best model in C1-negative patients

The KIR locus is divided into a centromeric and a telomeric region, each bearing variable KIR gene content motifs designated as cen-A, cen-B, tel-A, and tel-B. The B-motifs contain one or more of the seven KIR B-haplotype specific genes. It has been shown by Cooley *et al*, that C1-positive, but not C1-negative patients benefit from transplantation with donors who carry two or more B-motifs. We used the B-content calculator that can be found at http://www.ebi.ac.uk/ipd/kir/donor_b_content.html to assign the donors of our cohort accordingly to one of three groups based on KIR B-content—neutral (n = 142), better (n = 40) or best (n = 18)—and analysed the impact of this classification on the transplantation outcome of the C1-negative patients. As case numbers in the “best” group were low, we decided to combine the “better” and the “best” donors into the “better/best” group. Multivariate analyses showed that in 9/10 matched transplantations, “better/best” donors may improve DFS (HR = 0.39, CI = 0.15–0.99, p = 0.048), but had no significant effect on OS (HR = 0.69, CI = 0.22–2.21, p = 0.52), RI (HR = 0.48, CI = 0.07–3.29, p = 0.46), or TRM (HR = 0.76, CI = 0.23–2.49, p = 0.65). In 10/10 matched transplant pairs, however, the “better/best” KIR donor status had no effect on transplantation outcome (OS: HR = 0.98, CI = 0.46–2.10, p = 0.95; DFS: HR = 1.13, CI = 0.63–2.02, p = 0.69; RI: HR = 1.60, CI = 0.72–3.55, p = 0.25; TRM: HR = 0.80, CI = 0.17–3.80, p = 0.78).

### Proposed algorithm for the selection for donors for C1-negative patients

**Fig 21:**
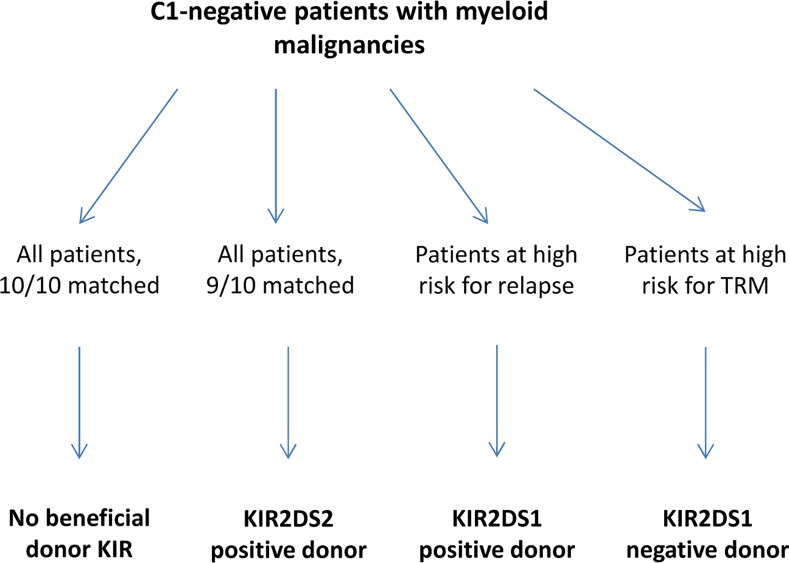
Proposed KIR dependant search algorithm for C1-negative patients with myeloid malignancies.

If our findings prove to be correct, and can be confirmed by others, then a possible selection algorithm of donors for C1-negative patients with myeloid malignancies could be as displayed in Fig 21.

## Discussion

The missing ligand model is based on the premise that underrepresentation of inhibiting signals will increase donor NK-alloreactivity against remaining malignant cells in the patient. As already described in the results section, all patients and donors within our cohort where positive for at least one inhibitory KIR that binds to HLA-C ligands. These results are consistent with the KIR-frequencies in Caucasoid and German populations provided by allelefrequencies.net[47]. We subsequently investigated if there was any effect of the missing ligand model on unrelated HSCT outcome. Deficiency for the C1-ligand had significantly negative effects on all analysed survival endpoints (OS, DFS, RI, and TRM). In contrast, C2-negative patient status did not affect any of these survival endpoints. Our findings are supported by studies done by Fischer *et al*.[48] (patients with CML), Giebel *et al*.[49] (various malignancies), and Hsu *et al*.[50] (AML and MDS) who also showed a negative impact of patient C1-ligand absence in HSCT outcome. Subsequent analyses confirmed that these findings applied for patients with myeloid malignancies but not for lymphoproliferative diseases. The C1-negative patient group with myeloid malignancies was therefore identified as being at higher risk than C1-positive patients and those with lymphatic diseases. Our findings can be explained by an impaired donor derived NK-cell activity in C1-ligand negative patients. During immune reconstitution the first KIRs to be expressed are KIR2DL2 and KIR2DL3, which recognize C1-ligands[48]. The early onset of donor NK-cells in these patients is consequently non-reactive against C1-ligand negative recipient cells. Furthermore, C1-restricted NK-cells form bigger populations, react faster to interferon γ secretion, and degranulate CD107a more potently than C2-restricted NK cells[51;52]. Additionally, the development of NK-cell activity is dependent on a process called NK-cell licensing which is mediated by interaction with an inhibitory receptor and its ligand[27;53]. Missing inhibition fails NK-cell licensing and therefore the NK-cells remain hypo-responsive. Interestingly, non-licensed NK-cells react extremely hyper-responsive against malignant cells in the early post-transplantation phase[53]. The late occurrence of KIR2DL1 cell surface expression therefore may impair NK-cell activity in two ways: by the failure to take advantage of the hyper-responsive early phase, and by the prolonged hypo-responsiveness thereafter. A further difference between C1-KIR2DL2/3 and C2-KIR2DL1 interactions lies within the binding affinity of the receptors to their ligands. Herein, C1-KIR2DL2/3 interactions are considerably weaker than those between C2 and KIR2DL1[18;51;54;55]. A weak binding affinity may promote overwriting of inhibitory by activating signals and therefore make the NK-cell more potent in killing of malignant cells[18]. The dependence of the observed effect from disease group, however, may be explained by sophisticated defence mechanisms of lymphatic blasts against NK-cell activity. CLL-samples have been shown to produce high amounts of tolerogenic HLA-G1 which promotes the acquisition of inhibitory NK cell receptors[56]. Furthermore, CLL and ALL patients show a selective down-regulation of non-Bw4 HLA-molecules (HLA-A and -B molecules with the Bw4 motif are ligands for inhibitory KIR)[6]–a mechanism which impairs both, NK and T-cell activity. The already impaired immune response against malignant cells therefore seems not to be further influenced by the additional impairment caused by the absence of the C1-ligand.

Transplantation with B-haplotype positive donors has been shown to have beneficial effects on HSCT[28;29]. Since gene content in B-haplotypes is highly variable and the main difference to A-haplotypes is the presence of other activating KIRs than KIR2DS4, we focused our analysis on the effects of the remaining activating KIRs on transplantation outcome in the identified risk group. Overall survival and disease free survival were greatly improved upon transplantation with a KIR2DS2-positive donor in the single HLA-class I mismatched setting. Furthermore, again in the presence of a single HLA-class I mismatch, RI was significantly reduced in risk group patients who had received a graft from a KIR2DS1 -positive donor when compared to risk group patients who were transplanted with KIR2DS1-negative donor cells. However, transplantation with a donor who carried KIR2DS1, resulted in a higher TRM, respectively. These results are surprising, if one considers that activated NK cells have been shown to mediate graft-versus-leukemia effects (i.e. lower relapse rates) in conjunction with a suppression of graft-versus-host disease (a major cause for higher TRM)[8;9]. Therefore, we expected lower relapse rates without an increase in TRM rates. Given that all patients in our study cohort received T-cell repleted grafts, a T-cell mediated effect may explain these controversial findings. Indeed, it has been shown that activated NK cells are able to directly communicate with CD4 positive T-cells via OX40—OX40 ligand interactions[57] and T-cells which have been activated by OX40 ligand binding have been detected in peripheral inflammatory sites of GvHD[58].

To our knowledge, we are the first to describe a beneficial effect of KIR2DS2 positive donors in HSCT, whereas a beneficial effect of transplantation with KIR2DS1-positive donors has been already described by several study groups[36;37;59]. However, in these studies the impact of HLA-matching grade in this context had not been taken into account. Taking advantage of a large multicentre study cohort of 1446 transplant pairs we were able to identify the 9/10 HLA-class I mismatched transplants as the decisive patient subgroup. We assume that, since activating signalling by KIR2DS1-C2 interaction is dominated by inhibiting KIR2DL1-C2[18;36], the effect of KIR2DS1-expressing donor NK-cells may be neutralized in 10/10 matched transplantations. In 9/10 matched transplant pairs, however, this domination could be shifted towards NK-cell activation by the stimulation of additional activating NK-receptors besides KIR, which enables KIR2DS1 (and maybe KIR2DS5) positive NK-cells to unfold their anti-leukemic reactivity.

There is some evidence for the potential of KIR2DS2-expressing NK-cells to control inflammatory processes[19], which would explain the positive influence of these cells on overall survival and disease free survival, but not on relapse incidence. Admittedly, the positive impact of KIR2DS2 in a patient group, which is negative for this receptors ligand seems to be hard to explain. However, the interaction potential of this receptor to ligands of the C1-group is yet not fully proven. Binding between KIR2DS2 and the C1-molecule HLA-C*16:01 but not with further C1-ligands has been shown to be significant[19]. Additionally, there is evidence of peptide-dependant binding capacities between KIR2DS2 and genes of the HLA-A*11 group[60]. There may be additional ligands for this receptor which enable its activation, even in C1-negative patients.

Cooley *et al*. investigated the effect on donor KIR B-haplotype status on transplantation outcome in unrelated matched HSCT in AML patients with respect to the centromeric and telomeric A- and B-haplotype motifs on the KIR locus[30]. According to their B-motif content, donors were grouped into “better/best” and “neutral”, wherein the patients profited from transplantation with “better/best” donors. However, they could only show a significant effect of this model in C1-positive, but not C1-negative donors. We identified KIR2DS2 as the KIR with the most important effect on HSCT outcome in C1-negative patients with myeloid malignancies, yet the KIR B-motif model implies that KIR2DS2 may or may not be present in the “better/best” group, so we hypothesized that this model would blur the effects which we have found by analysing the activating KIRs separately. Indeed, the “better/best” donors showed no beneficial effect on transplantation outcome in the C1-negative patients. Hence, the KIR B-motif model indeed showed a diminishing impact on C1-negative patients and therefore for this patient group complete KIR locus typing seems to bear no advantage. Instead, our results recommend donor typing for KIR2DS2 only (and possibly also for KIR2DS1).

In summary, our study shows, that activating signals derived from KIR2DS1 and possibly KIR2DS5 can overcome the impaired NK-cell response in the C1-negative risk patient group, but also provoke the risk for TRM. These findings provide new insights for the selection of a suitable donor for the C1 negative risk patient group: for patients who are at high risk for relapse a KIR2DS1 donor positive may be an adequate choice, whereas a graft which is negative for this KIR may be an option when a high probability for treatment related complications is anticipated. The most important finding of our study, however, is the association of donor KIR2DS2 positivity with a better overall outcome after HSCT in the C1-negative risk patient group.

C1-negative patients with myeloid malignancies may therefore benefit from the inclusion of KIR2DS1- and more importantly KIR2DS2-genotyping in the search algorithm. Our findings certainly need to be confirmed by further studies in an independent cohort and also possibly by a prospective study, prior to conduct changes in the search algorithm.

### Limitations

Our approach was to identify beneficial donor KIRs in the relatively small group of C1-negative patients (14% of all patients in our cohort). In this context, small subgroups were inevitable. However, while our study was limited by small sample sizes it nonetheless demonstrated statistically significant findings with a robust effect size.

## Supporting Information

S1 Fig Univariate OS analysis in C1-negative patients (missing C1 ligand) vs. C1-positive patients who were transplanted with KIR2DL2 or KIR2DL3 positive donors. Dashed red line: C1-negative patients (n = 200), fine red lines: corresponding confidence intervals. Solid black line: C1-positive patients (n = 1239), fine black lines: corresponding confidence intervals. p = 0.0014.
(TIFF)

S2 Fig Univariate DFS analysis in C1-negative patients (missing C1 ligand) vs. C1-positive patients who were transplanted with KIR2DL2 or KIR2DL3 positive donors. Dashed red line: C1-negative patients (n = 200), fine red lines: corresponding confidence intervals. Solid black line: C1-positive patients (n = 1239), fine black lines: corresponding confidence intervals. p = 0.006.
(TIFF)

S3 Fig Univariate RI analysis in C1-negative patients (missing C1 ligand) vs. C1-positive patients who were transplanted with KIR2DL2 or KIR2DL3 positive donors. Dashed red line: C1-negative patients (n = 200), fine red lines: corresponding confidence intervals. Solid black line: C1-positive patients (n = 1239), fine black lines: corresponding confidence intervals. p = 0.04.
(TIFF)

S4 Fig Univariate TRM analysis in C1-negative patients (missing C1 ligand) vs. C1-positive patients who were transplanted with KIR2DL2 or KIR2DL3 positive donors. Dashed red line: C1-negative patients (n = 200), fine red lines: corresponding confidence intervals. Solid black line: C1-positive patients (n = 1239), fine black lines: corresponding confidence intervals. p = 0.06.
(TIFF)

S5 Fig Univariate OS analysis in C2-negative (missing C2 ligand) vs. C2-positive patients who were transplanted with KIR2DL1-positive donors. Dashed red line: C2-negative patients (n = 823), fine red lines: corresponding confidence intervals. Solid black line: C2-positive patients (n = 558), fine black lines: corresponding confidence intervals. p = 0.08.
(TIFF)

S6 Fig Univariate DFS analysis in C2-negative (missing C2 ligand) vs. C2-positive patients who were transplanted with KIR2DL1-positive donors. Dashed red line: C2-negative patients (n = 823), fine red lines: corresponding confidence intervals. Solid black line: C2-positive patients (n = 558), fine black lines: corresponding confidence intervals. p = 0.11.
(TIFF)

S7 Fig Univariate RI analysis in C2-negative (missing C2 ligand) vs. C2-positive patients who were transplanted with KIR2DL1-positive donors. Dashed red line: C2-negative patients (n = 823), fine red lines: corresponding confidence intervals. Solid black line: C2-positive patients (n = 558), fine black lines: corresponding confidence intervals. p = 0.47.
(TIFF)

S8 Fig Univariate TRM analysis in C2-negative (missing C2 ligand) vs. C2-positive patients who were transplanted with KIR2DL1-positive donors. Dashed red line: C2-negative patients (n = 823), fine red lines: corresponding confidence intervals. Solid black line: C2-positive patients (n = 558), fine black lines: corresponding confidence intervals. p = 0.30.
(TIFF)

S9 Fig Univariate OS analysis in C1-negative patients with myeloid malignancies who were transplanted with KIR2DS2 positive donors vs. KIR2DS2 negative donors. Solid black line: donor KIR2DS2-negative, fine black lines: corresponding confidence intervals. Dashed red line: donor KIR2DS2-positive, fine red lines: corresponding confidence intervals. 10/10 matched transplant pairs, donor KIR2DS2-negative (n = 32) vs. donor KIR2DS2-positive (n = 45), p = 0.86.
(TIFF)

S10 Fig Univariate OS analysis in C1-negative patients with myeloid malignancies who were transplanted with KIR2DS2 positive donors vs. KIR2DS2 negative donors. Solid black line: donor KIR2DS2-negative, fine black lines: corresponding confidence intervals. Dashed red line: donor KIR2DS2-positive, fine red lines: corresponding confidence intervals. 9/10 matched transplant pairs, donor KIR2DS2-negative (n = 22) vs. donor KIR2DS2-positive (n = 18), p = 0.04.
(TIFF)

S11 Fig DFS analysis in C1-negative patients with myeloid malignancies who were transplanted with KIR2DS2 positive donors vs. KIR2DS2 negative donors. Solid black line: donor KIR2DS2-negative, fine black lines: corresponding confidence intervals. Dashed red line: donor KIR2DS2-positive, fine red lines: corresponding confidence intervals. 10/10 matched transplant pairs, donor KIR2DS2-negative (n = 32) vs. donor KIR2DS2-positive (n = 45), p = 0.88.
(TIFF)

S12 Fig DFS analysis in C1-negative patients with myeloid malignancies who were transplanted with KIR2DS2 positive donors vs. KIR2DS2 negative donors. Solid black line: donor KIR2DS2-negative, fine black lines: corresponding confidence intervals. Dashed red line: donor KIR2DS2-positive, fine red lines: corresponding confidence intervals. 9/10 matched transplant pairs, donor KIR2DS2-negative (n = 22) vs. donor KIR2DS2-positive (n = 18), p = 0.05.
(TIFF)

S13 Fig Univariate RI analysis in C1-negative patients with myeloid malignancies who were transplanted with KIR2DS1 positive vs. KIR2DS1 negative donors. Solid black line: donor KIR2DS1 negative, fine black lines: corresponding confidence intervals. Dashed red line: donor KIR2DS1 positive, fine red lines: corresponding confidence intervals. Donor KIR2DS1-negative (n = 72) vs. donor KIR2DS1-positive (n = 45), p = 0.008.
(TIFF)

S14 Fig Univariate RI analysis in C1-negative patients with myeloid malignancies who were transplanted with KIR2DS2 positive vs. KIR2DS2 negative donors. Solid black line: donor KIR2DS2 negative, fine black lines: corresponding confidence intervals. Dashed red line: donor KIR2DS2 positive, fine red lines: corresponding confidence intervals. Donor KIR2DS2-negative (n = 54) vs. donor KIR2DS2-positive (n = 63), p = 0.33.
(TIFF)

S15 Fig Univariate RI analysis in C1-negative patients with myeloid malignancies who were transplanted with KIR2DS5 positive vs. KIR2DS5 negative donors. Solid black line: donor KIR2DS5 negative, fine black lines: corresponding confidence intervals. Dashed red line: donor 5 positive, fine red lines: corresponding confidence intervals. Donor KIR2DS5-negative (n = 83) vs. donor KIR2D5-positive (n = 34), p = 0.05.
(TIFF)

S16 Fig Univariate RI analysis in C1-negative patients with myeloid malignancies who were transplanted with KIR3DS1 positive vs. KIR3DS1 negative donors. Solid black line: donor KIR3DS1 negative, fine black lines: corresponding confidence intervals. Dashed red line: donor KIR3DS1 positive, fine red lines: corresponding confidence intervals. Donor KIR3DS1-negative (n = 72) vs. donor KIR3DS1-positive (n = 45), p = 0.06.
(TIFF)

S17 Fig Univariate TRM analysis in C1-negative patients with myeloid malignancies who were transplanted with KIR2DS1 positive vs. KIR2DS1 negative donors. Solid black line: donor KIR2DS1 negative, fine black lines: corresponding confidence intervals. Dashed red line: donor KIR2DS1 positive, fine red lines: corresponding confidence intervals. Donor KIR2DS1-negative (n = 72) vs. donor KIR2DS1-positive (n = 45), p = 0.06.
(TIFF)

S18 Fig Univariate TRM analysis in C1-negative patients with myeloid malignancies who were transplanted with KIR2DS5 positive vs. KIR2DS5 negative donors. Solid black line: donor KIR2DS5 negative, fine black lines: corresponding confidence intervals. Dashed red line: donor KIR2DS5 positive, fine red lines: corresponding confidence intervals. Donor KIR2DS5-negative (n = 83) vs. donor KIR2D5-positive (n = 34), p = 0.45.
(TIFF)

S19 Fig Univariate TRM analysis in C1-negative patients with myeloid malignancies who were transplanted with KIR2DS2 positive vs. KIR2DS2 negative donors. Solid black line: donor KIR2DS2 negative, fine black lines: corresponding confidence intervals. Dashed red line: donor KIR2DS2 positive, fine red lines: corresponding confidence intervals. Donor KIR2DS2-negative (n = 54) vs. donor KIR2DS2-positive (n = 63), p = 0.001.
(TIFF)

S20 Fig Univariate TRM analysis in C1-negative patients with myeloid malignancies who were transplanted with KIR3DS1 positive vs. KIR3DS1 negative donors. Solid black line: donor KIR3DS1 negative, fine black lines: corresponding confidence intervals. Dashed red line: donor KIR3DS1 positive, fine red lines: corresponding confidence intervals. Donor KIR3DS1-negative (n = 72) vs. donor KIR3DS1-positive (n = 45), p = 0.35.
(TIFF)

S1 Table (DOCX)

S2 Table (DOCX)

S3 Table (DOCX)

S4 Table (DOCX)

## Abbreviations

AL: Unclassified acute leukemia
ALL: Acute lymphoblastic leukemia
AML: Acute myeloid leukemia
CI: Confidence interval
CLL: Chronic lymphoblastic leukemia
CML: Chronic myeloid leukemia
DFS: Disease free survival
GvHD: Graft-versus-host disease
GvL: Graft-versus-leukemia
HLA: Human Leukocyte Antigen
HR: Hazard ratio
HSCT: Hematopoietic stem cell transplantation
KIR: Killer cell immunoglobulin-like receptors
MDS: Myelodysplastic syndrome
MM: Multiple myeloma
NK: Natural Killer cell
OS: Overall survival
RI: Relapse incidence
SBT: sequence based typing
TRM: Treatment related mortality
