# Supplementary figures and images for: Impact of Donor Activating KIR Genes on HSCT Outcome in C1-Ligand Negative Myeloid Disease Patients Transplanted with Unrelated Donors—A Retrospective Study
Source: PLoS One. 2017 Jan 20;12(1):e0169512. doi: 10.1371/journal.pone.0169512 (PMC5249182; doi:10.1371/journal.pone.0169512)

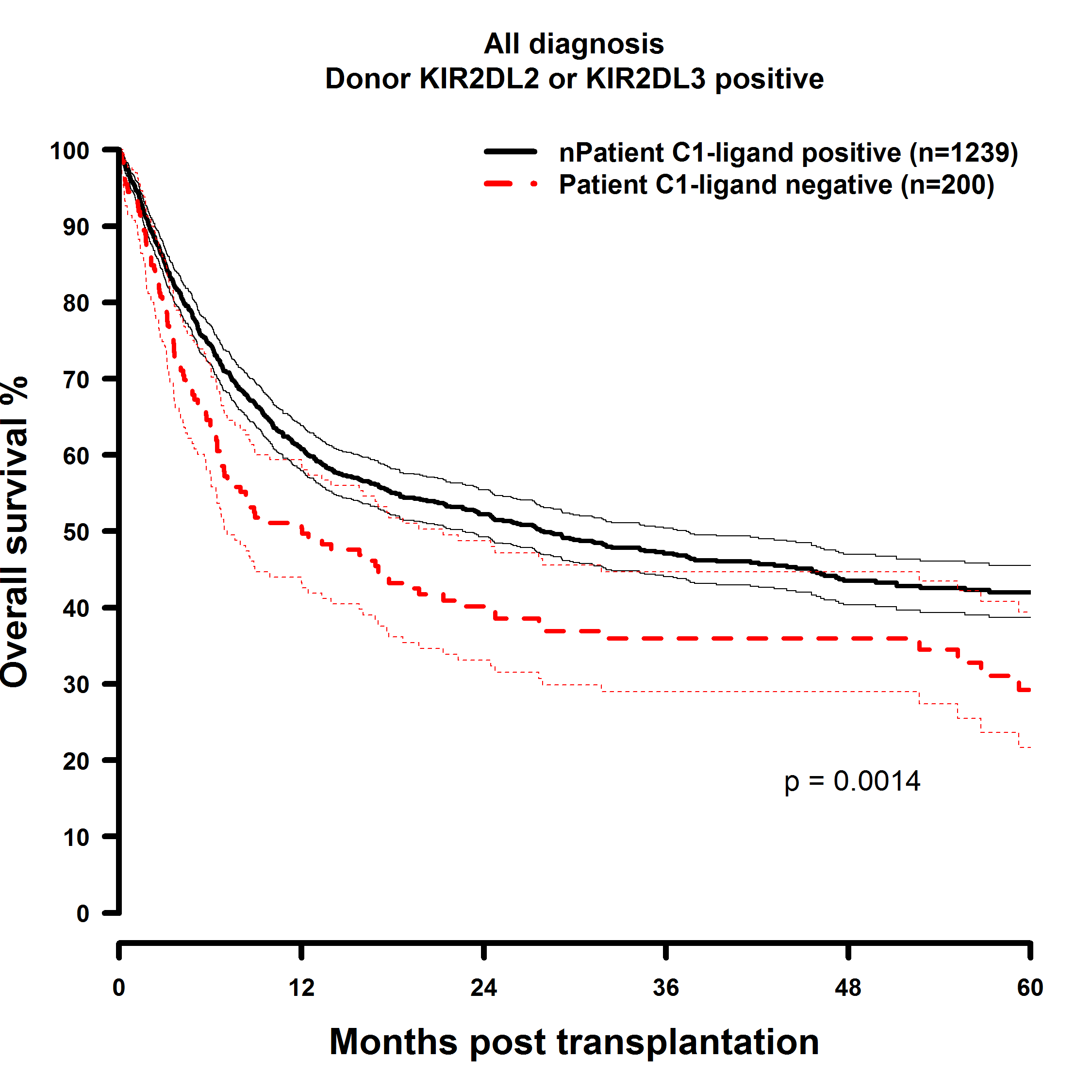

Supplement: S1 Fig — Dashed red line: C1-negative patients (n = 200), fine red lines: corresponding confidence intervals. Solid black line: C1-positive patients (n = 1239), fine black lines: corresponding confidence intervals. p = 0.0014. (TIFF) [file pone.0169512.s001.tiff]

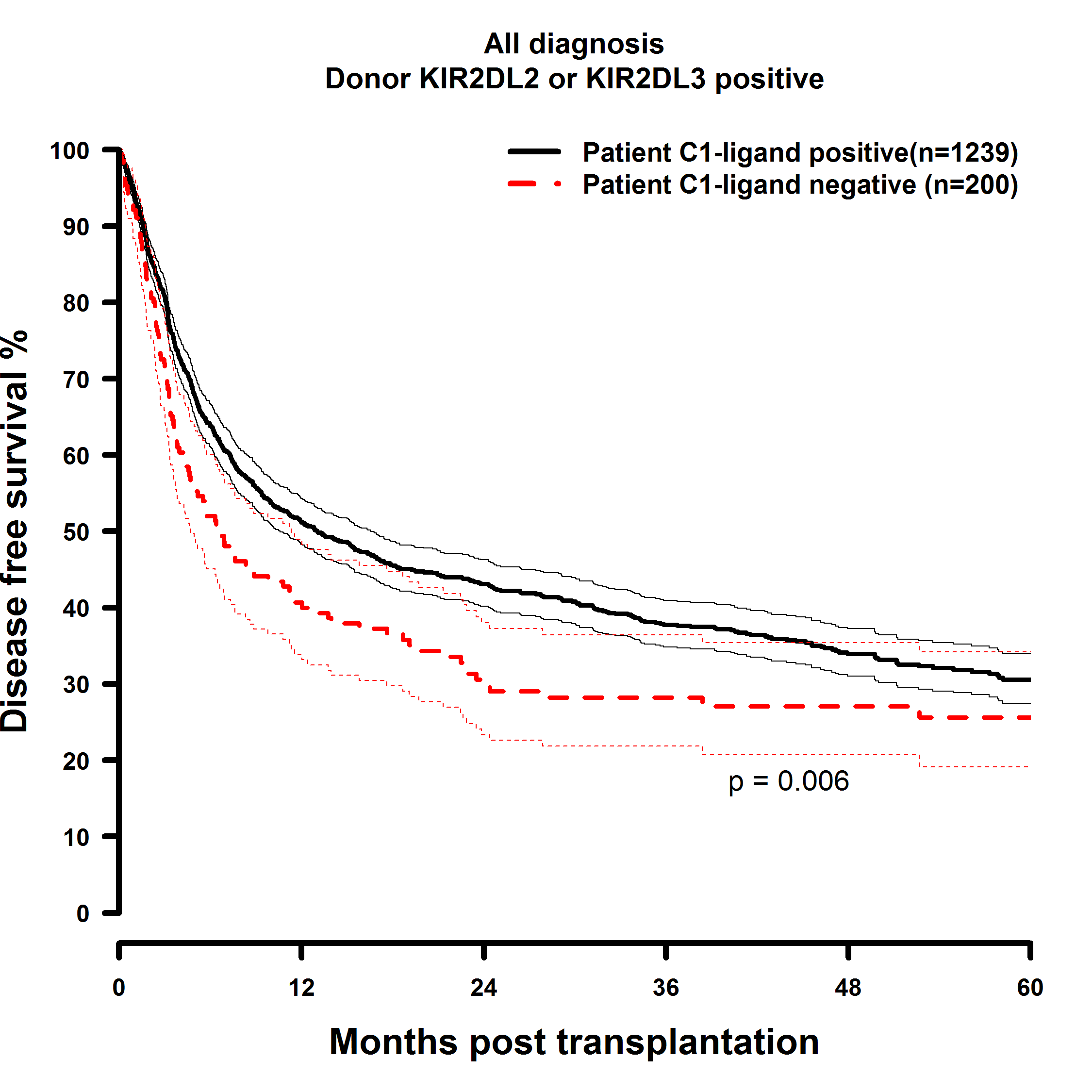

Supplement: S2 Fig — Dashed red line: C1-negative patients (n = 200), fine red lines: corresponding confidence intervals. Solid black line: C1-positive patients (n = 1239), fine black lines: corresponding confidence intervals. p = 0.006. (TIFF) [file pone.0169512.s002.tiff]

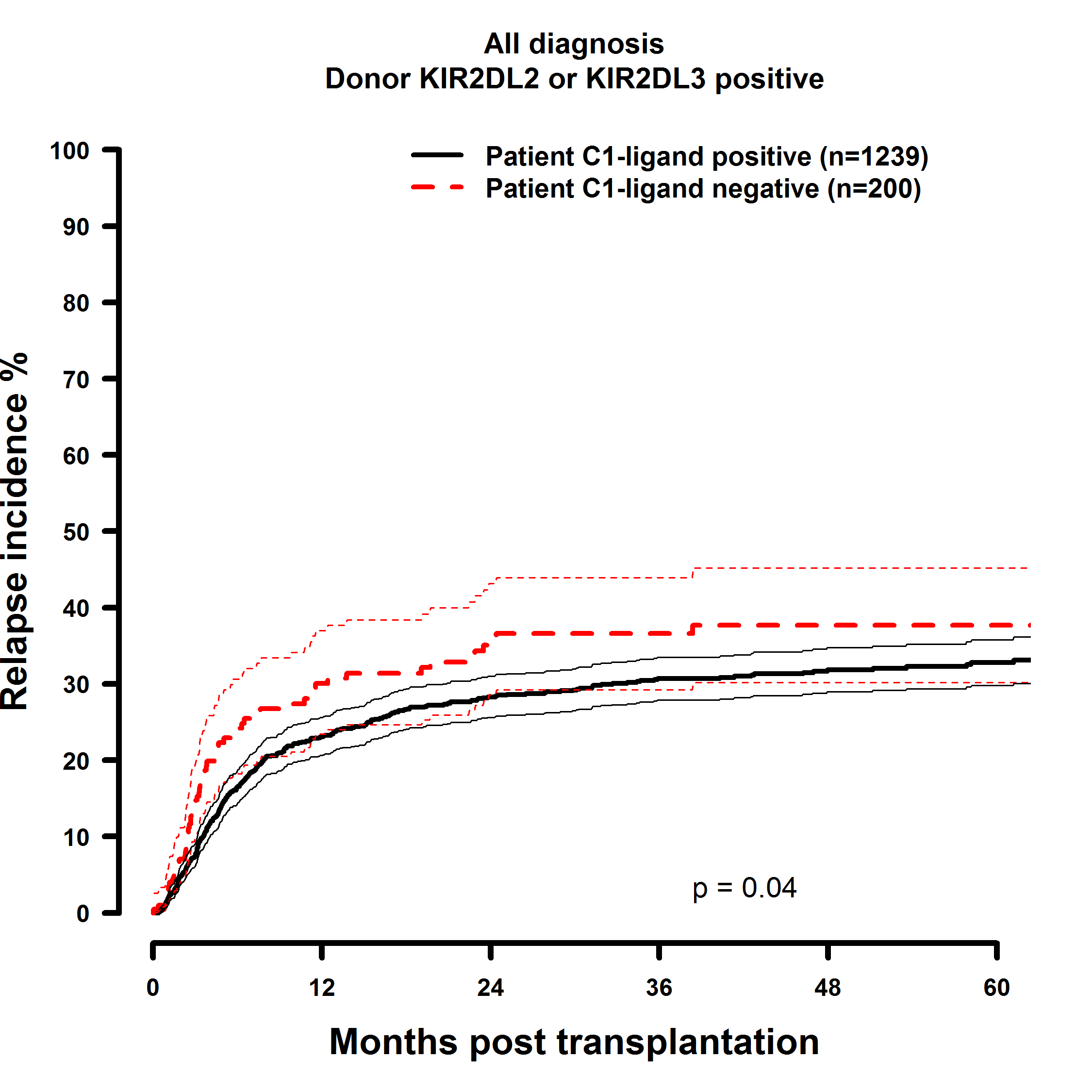

Supplement: S3 Fig — Dashed red line: C1-negative patients (n = 200), fine red lines: corresponding confidence intervals. Solid black line: C1-positive patients (n = 1239), fine black lines: corresponding confidence intervals. p = 0.04. (TIFF) [file pone.0169512.s003.tiff]

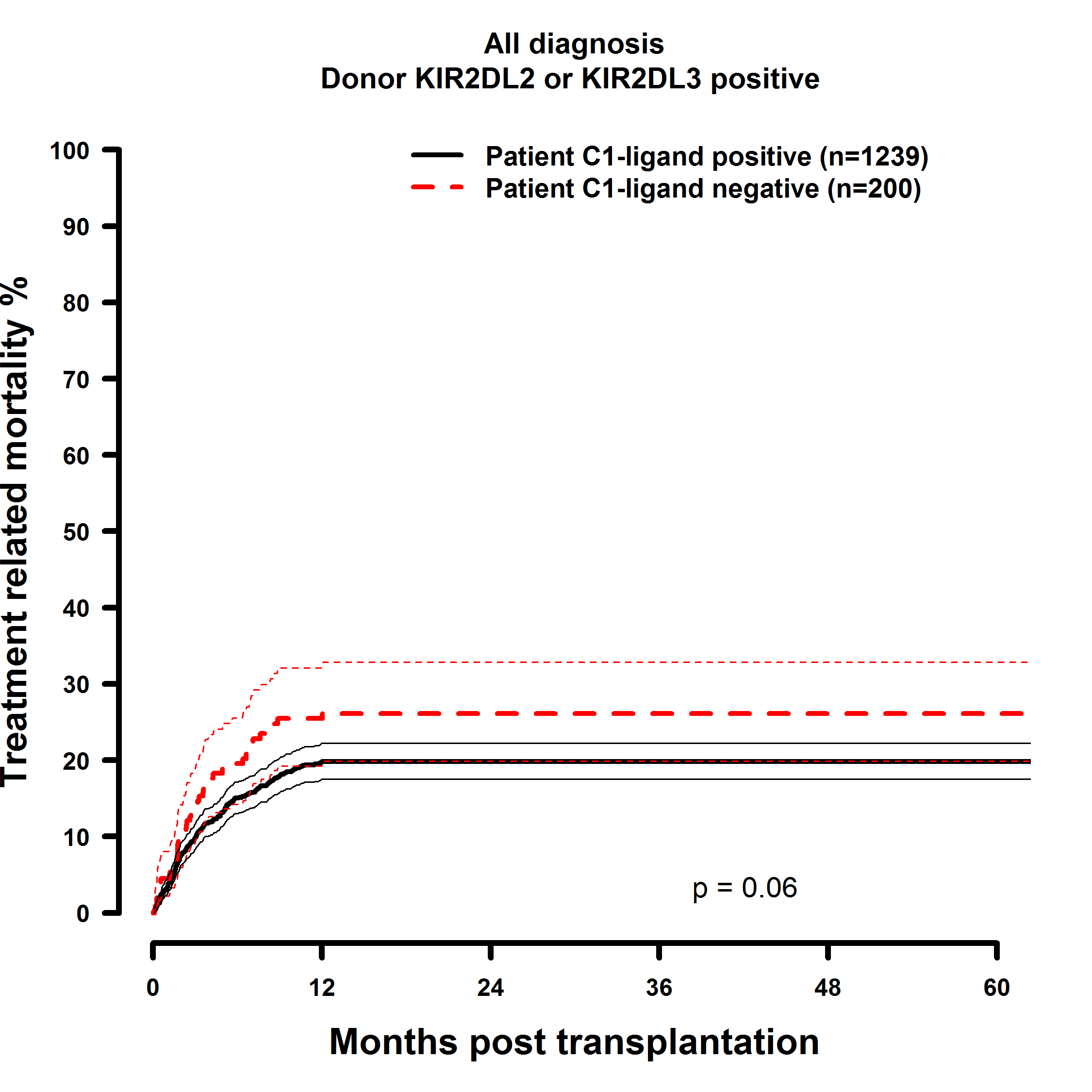

Supplement: S4 Fig — Dashed red line: C1-negative patients (n = 200), fine red lines: corresponding confidence intervals. Solid black line: C1-positive patients (n = 1239), fine black lines: corresponding confidence intervals. p = 0.06. (TIFF) [file pone.0169512.s004.tiff]

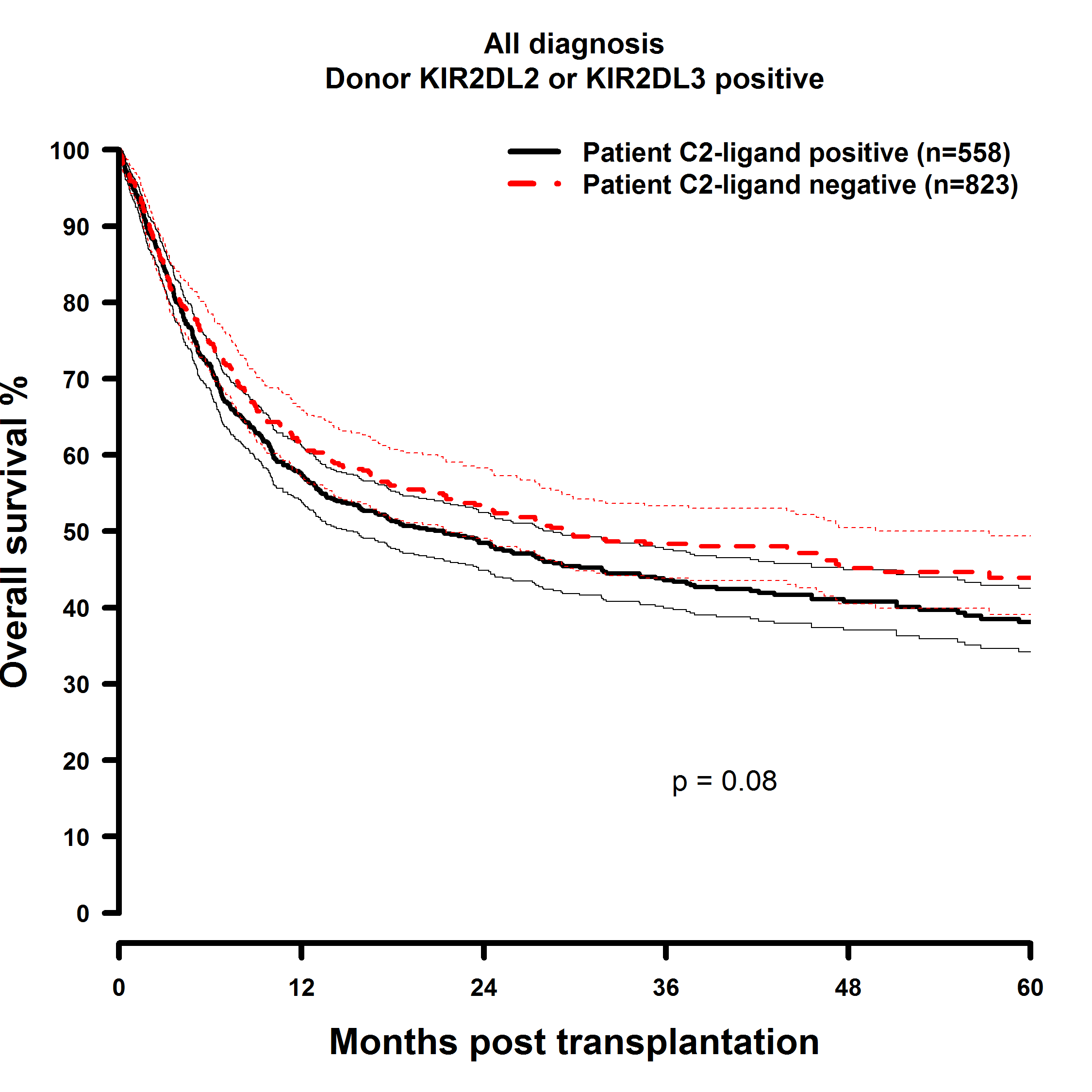

Supplement: S5 Fig — Dashed red line: C2-negative patients (n = 823), fine red lines: corresponding confidence intervals. Solid black line: C2-positive patients (n = 558), fine black lines: corresponding confidence intervals. p = 0.08. (TIFF) [file pone.0169512.s005.tiff]

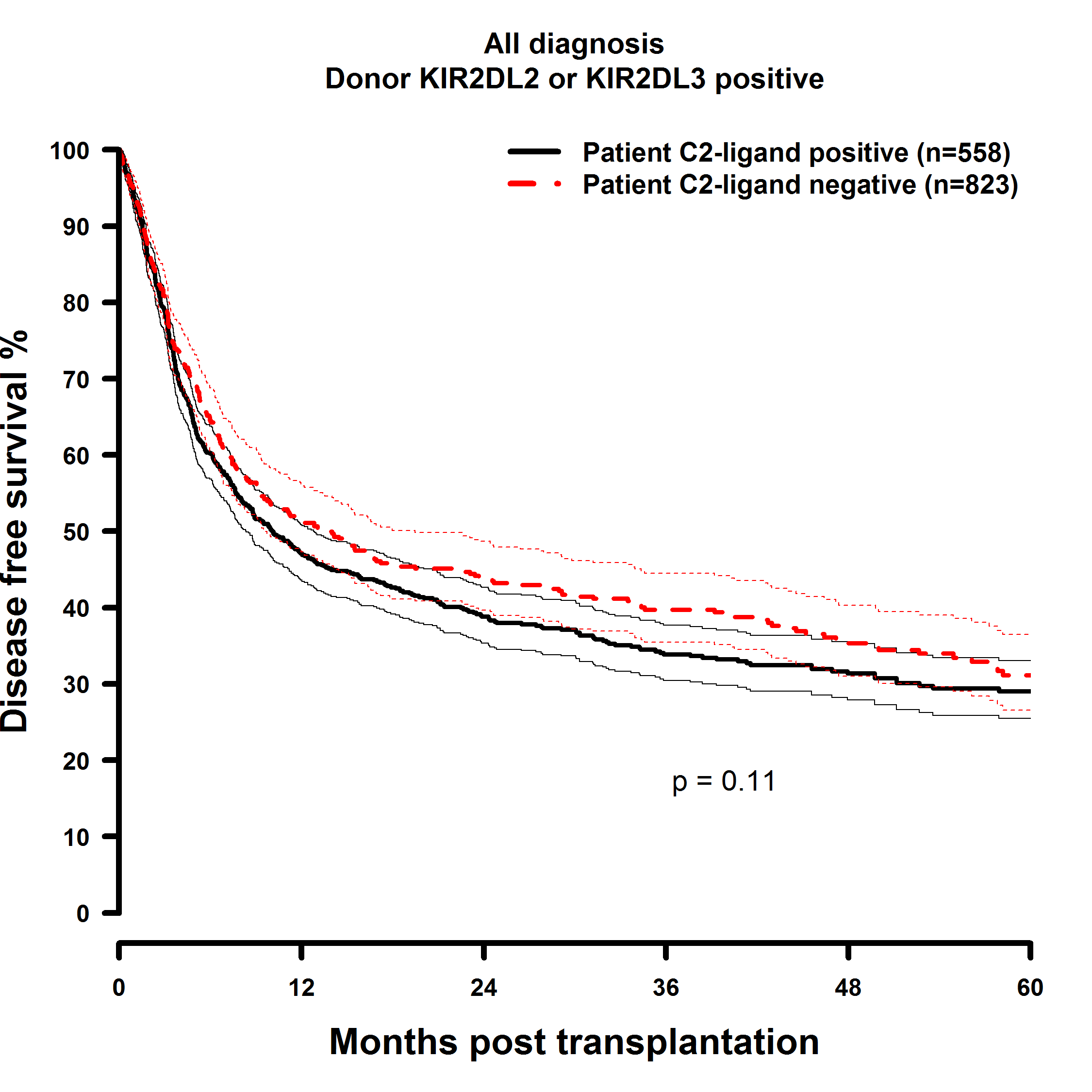

Supplement: S6 Fig — Dashed red line: C2-negative patients (n = 823), fine red lines: corresponding confidence intervals. Solid black line: C2-positive patients (n = 558), fine black lines: corresponding confidence intervals. p = 0.11. (TIFF) [file pone.0169512.s006.tiff]

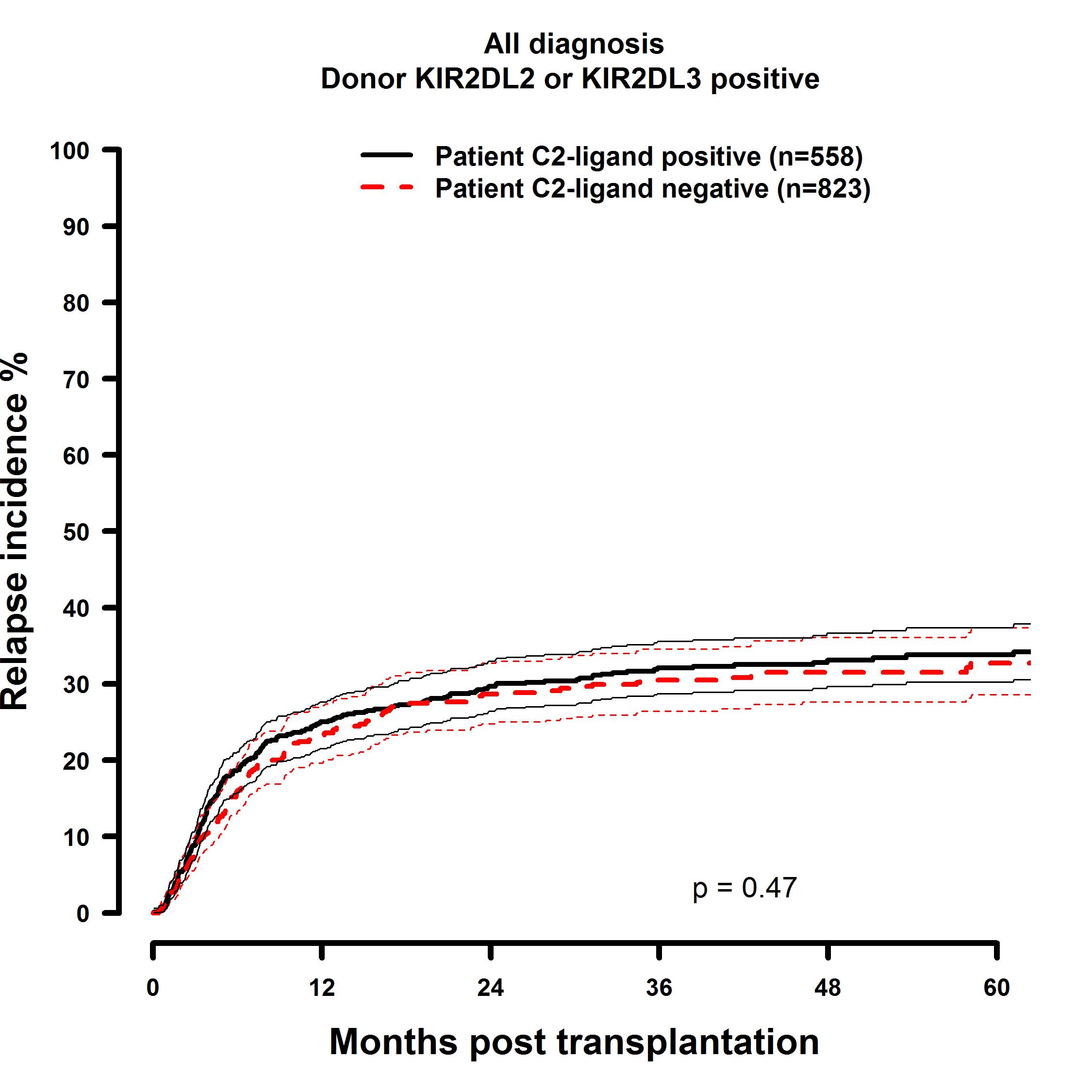

Supplement: S7 Fig — Dashed red line: C2-negative patients (n = 823), fine red lines: corresponding confidence intervals. Solid black line: C2-positive patients (n = 558), fine black lines: corresponding confidence intervals. p = 0.47. (TIFF) [file pone.0169512.s007.tiff]

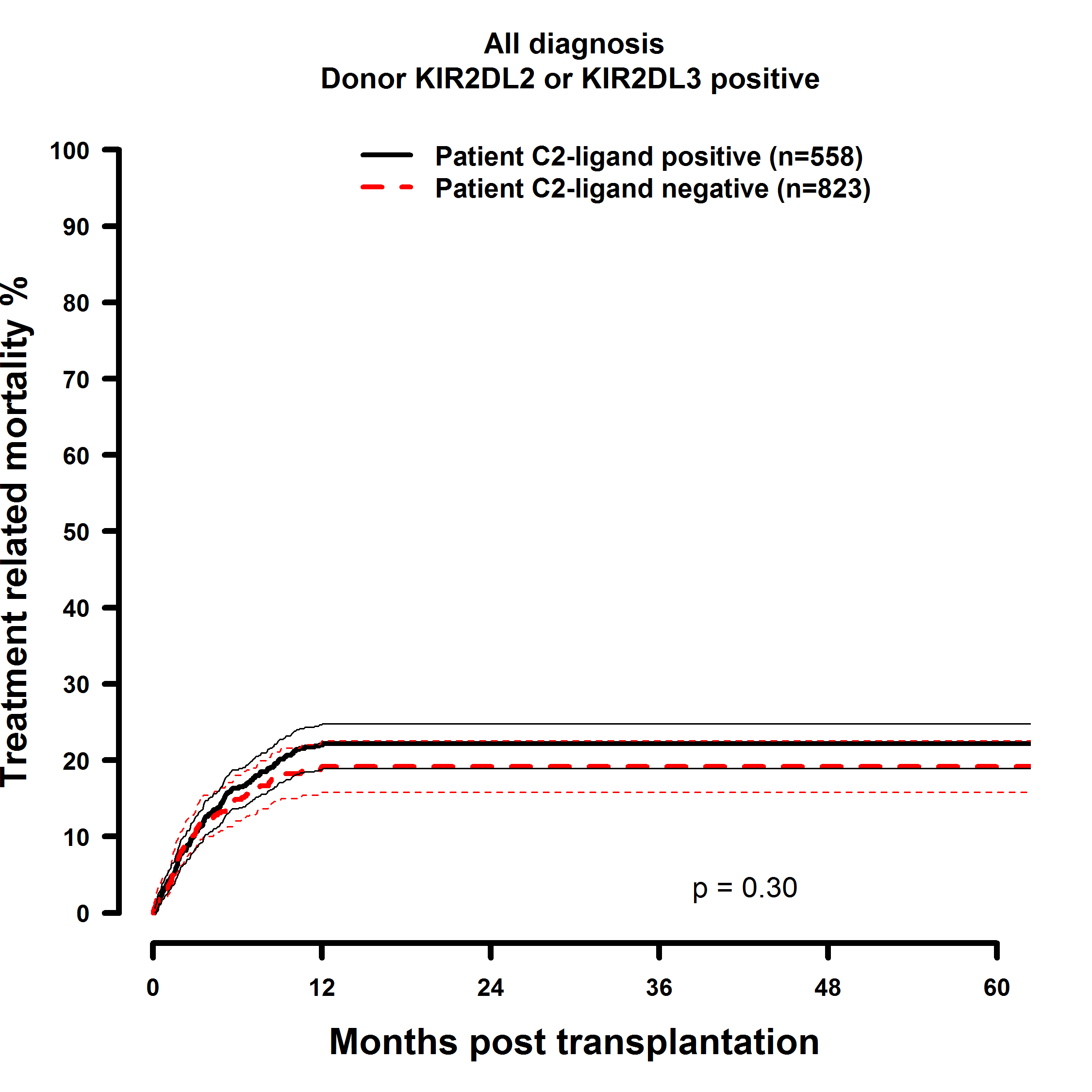

Supplement: S8 Fig — Dashed red line: C2-negative patients (n = 823), fine red lines: corresponding confidence intervals. Solid black line: C2-positive patients (n = 558), fine black lines: corresponding confidence intervals. p = 0.30. (TIFF) [file pone.0169512.s008.tiff]

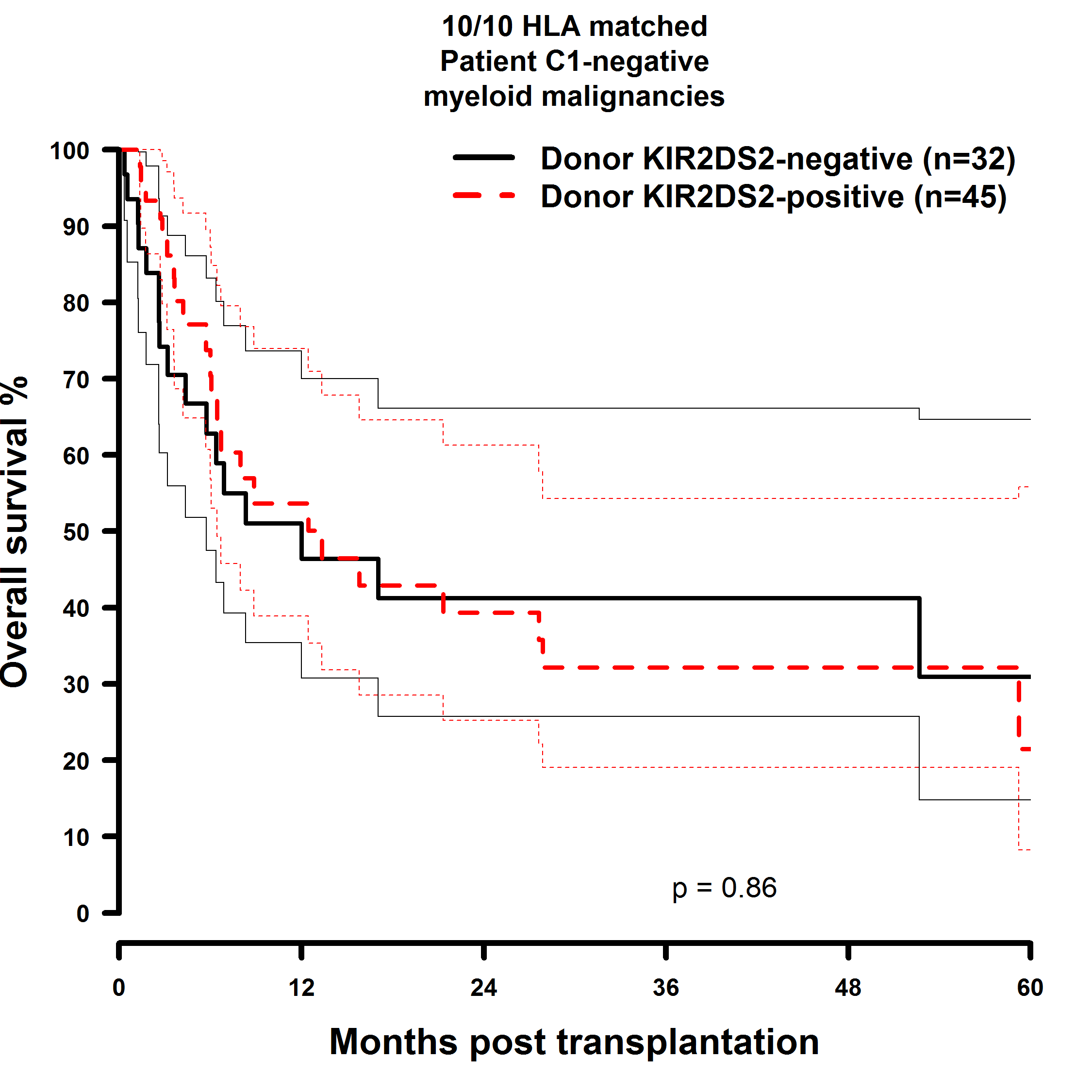

Supplement: S9 Fig — Solid black line: donor KIR2DS2-negative, fine black lines: corresponding confidence intervals. Dashed red line: donor KIR2DS2-positive, fine red lines: corresponding confidence intervals. 10/10 matched transplant pairs, donor KIR2DS2-negative (n = 32) vs. donor KIR2DS2-positive (n = 45), p = 0.86. (TIFF) [file pone.0169512.s009.tiff]

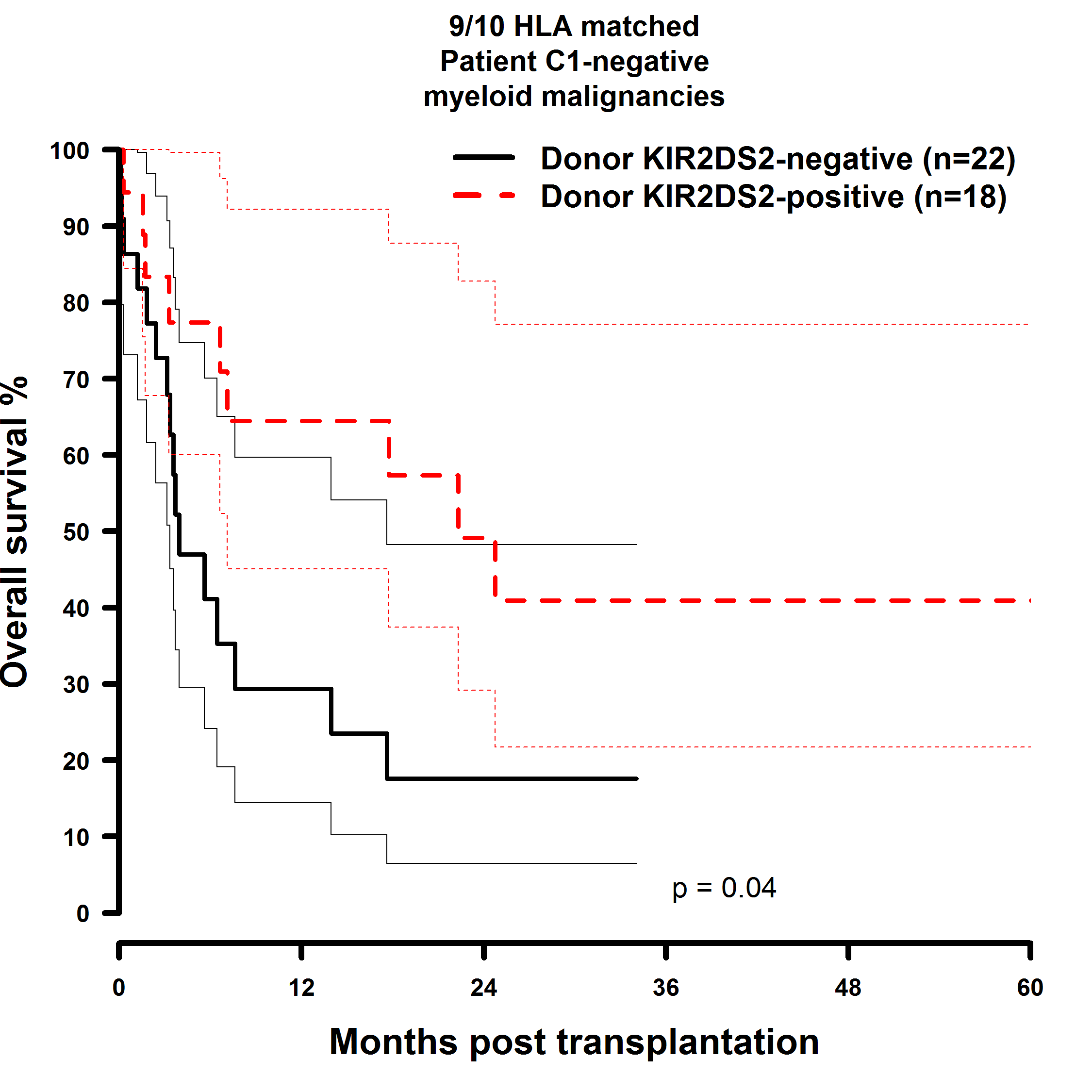

Supplement: S10 Fig — Solid black line: donor KIR2DS2-negative, fine black lines: corresponding confidence intervals. Dashed red line: donor KIR2DS2-positive, fine red lines: corresponding confidence intervals. 9/10 matched transplant pairs, donor KIR2DS2-negative (n = 22) vs. donor KIR2DS2-positive (n = 18), p = 0.04. (TIFF) [file pone.0169512.s010.tiff]

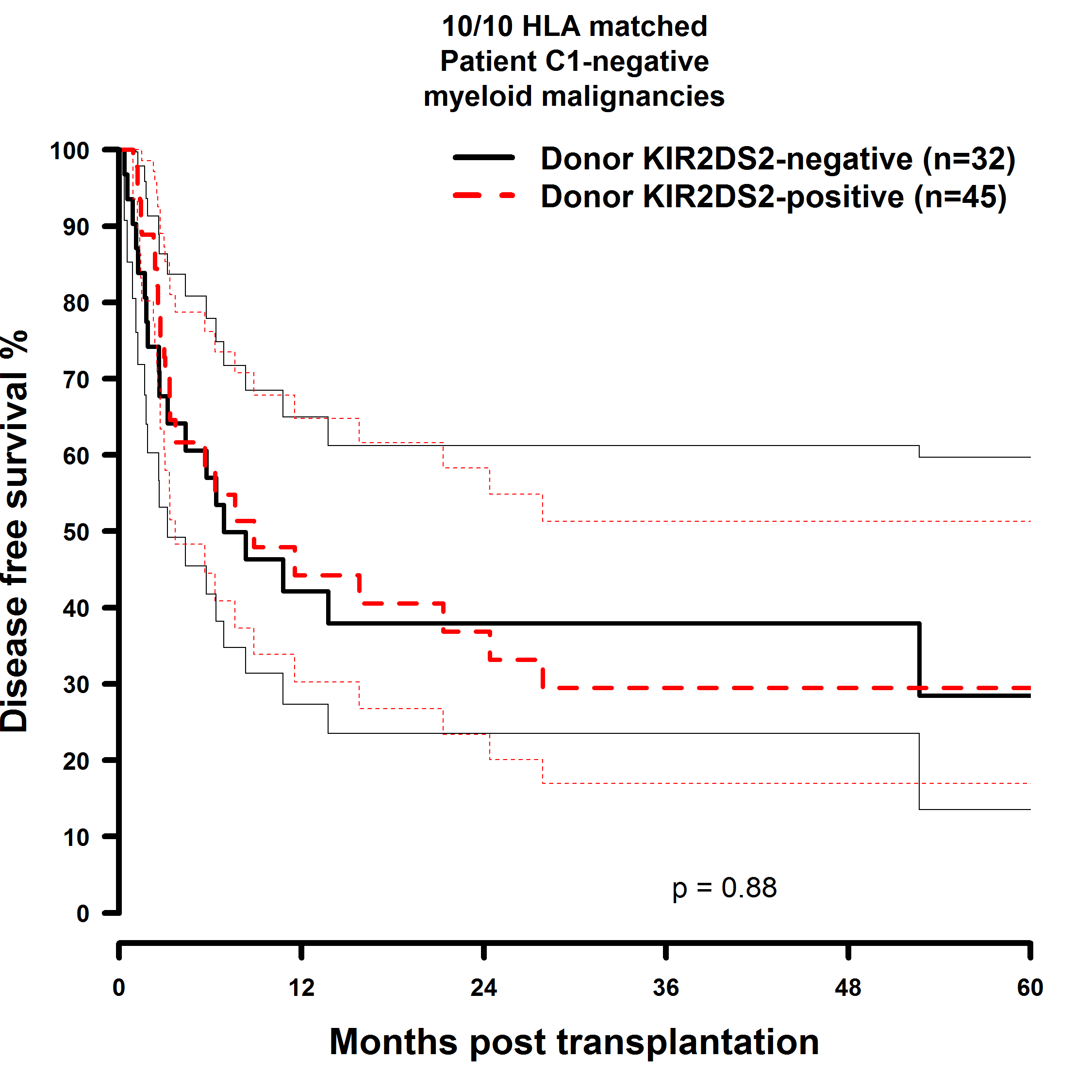

Supplement: S11 Fig — Solid black line: donor KIR2DS2-negative, fine black lines: corresponding confidence intervals. Dashed red line: donor KIR2DS2-positive, fine red lines: corresponding confidence intervals. 10/10 matched transplant pairs, donor KIR2DS2-negative (n = 32) vs. donor KIR2DS2-positive (n = 45), p = 0.88. (TIFF) [file pone.0169512.s011.tiff]

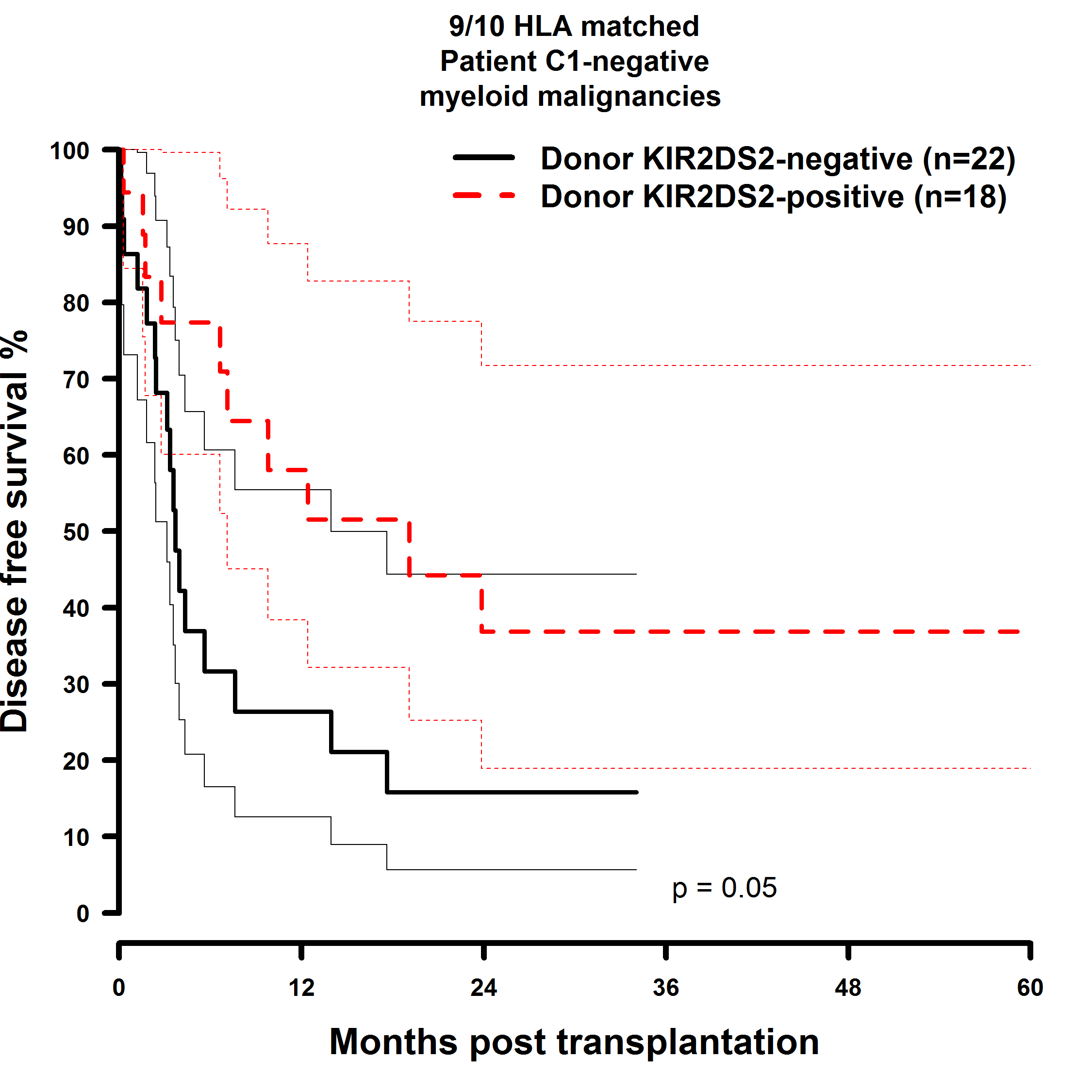

Supplement: S12 Fig — Solid black line: donor KIR2DS2-negative, fine black lines: corresponding confidence intervals. Dashed red line: donor KIR2DS2-positive, fine red lines: corresponding confidence intervals. 9/10 matched transplant pairs, donor KIR2DS2-negative (n = 22) vs. donor KIR2DS2-positive (n = 18), p = 0.05. (TIFF) [file pone.0169512.s012.tiff]

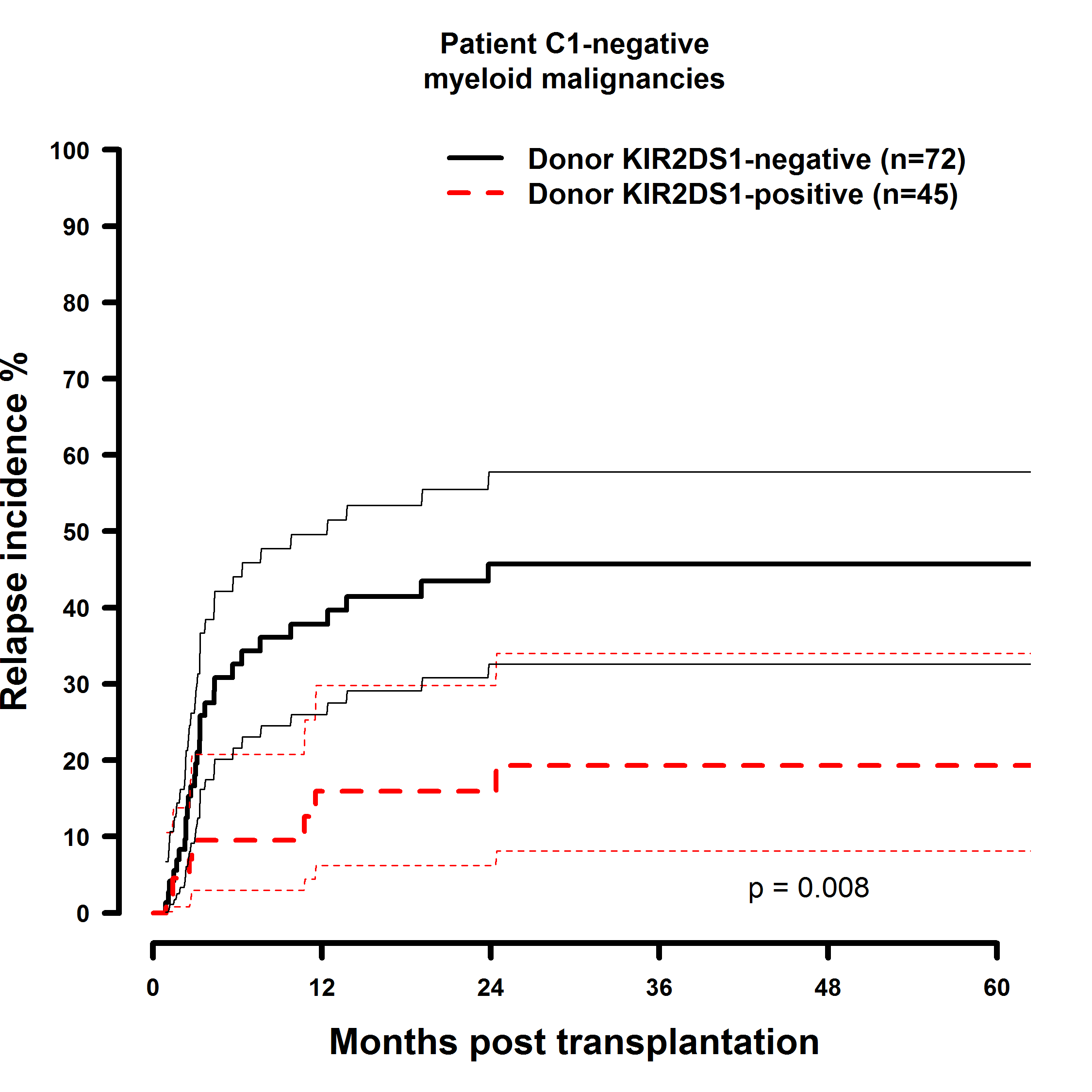

Supplement: S13 Fig — Solid black line: donor KIR2DS1 negative, fine black lines: corresponding confidence intervals. Dashed red line: donor KIR2DS1 positive, fine red lines: corresponding confidence intervals. Donor KIR2DS1-negative (n = 72) vs. donor KIR2DS1-positive (n = 45), p = 0.008. (TIFF) [file pone.0169512.s013.tiff]

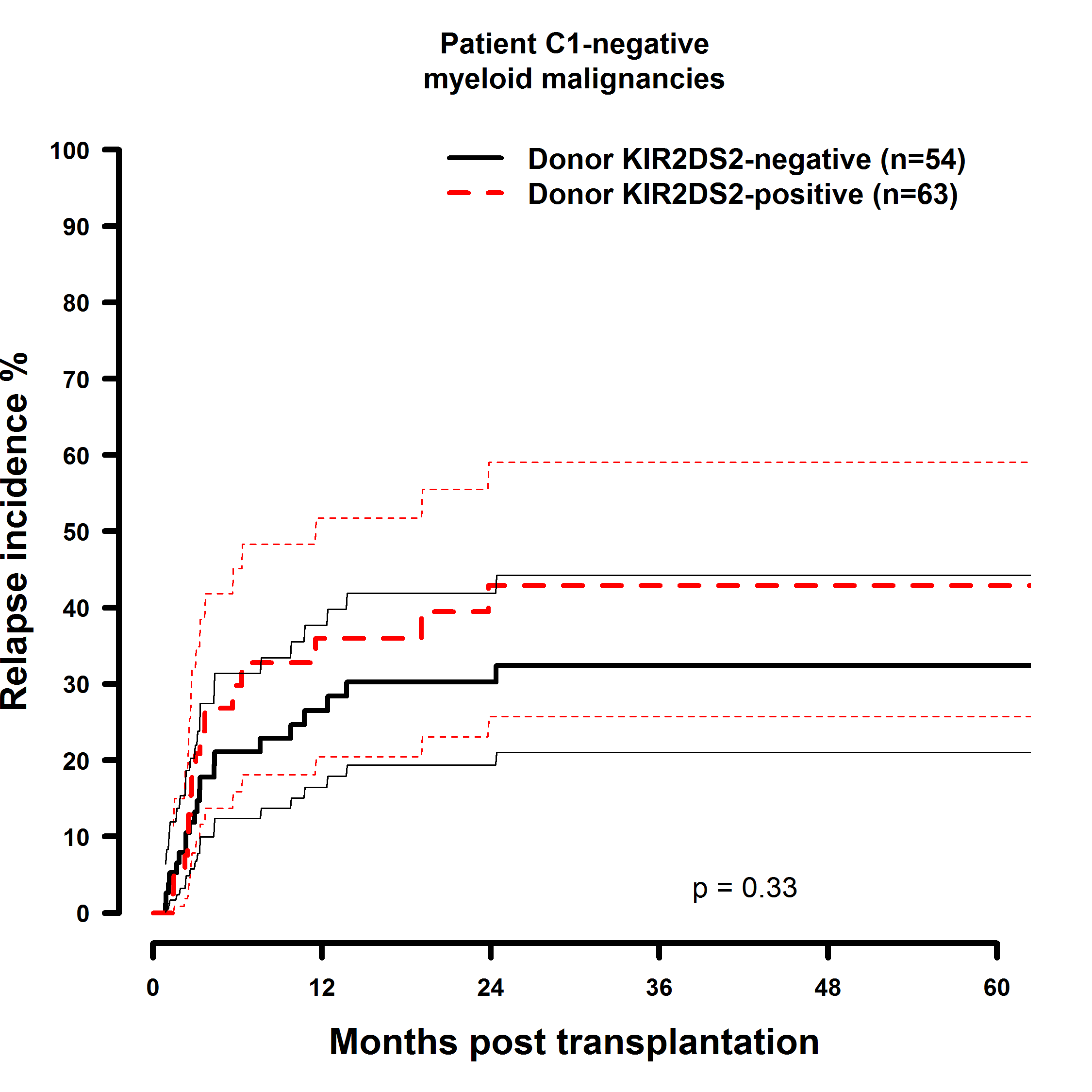

Supplement: S14 Fig — Solid black line: donor KIR2DS2 negative, fine black lines: corresponding confidence intervals. Dashed red line: donor KIR2DS2 positive, fine red lines: corresponding confidence intervals. Donor KIR2DS2-negative (n = 54) vs. donor KIR2DS2-positive (n = 63), p = 0.33. (TIFF) [file pone.0169512.s014.tiff]

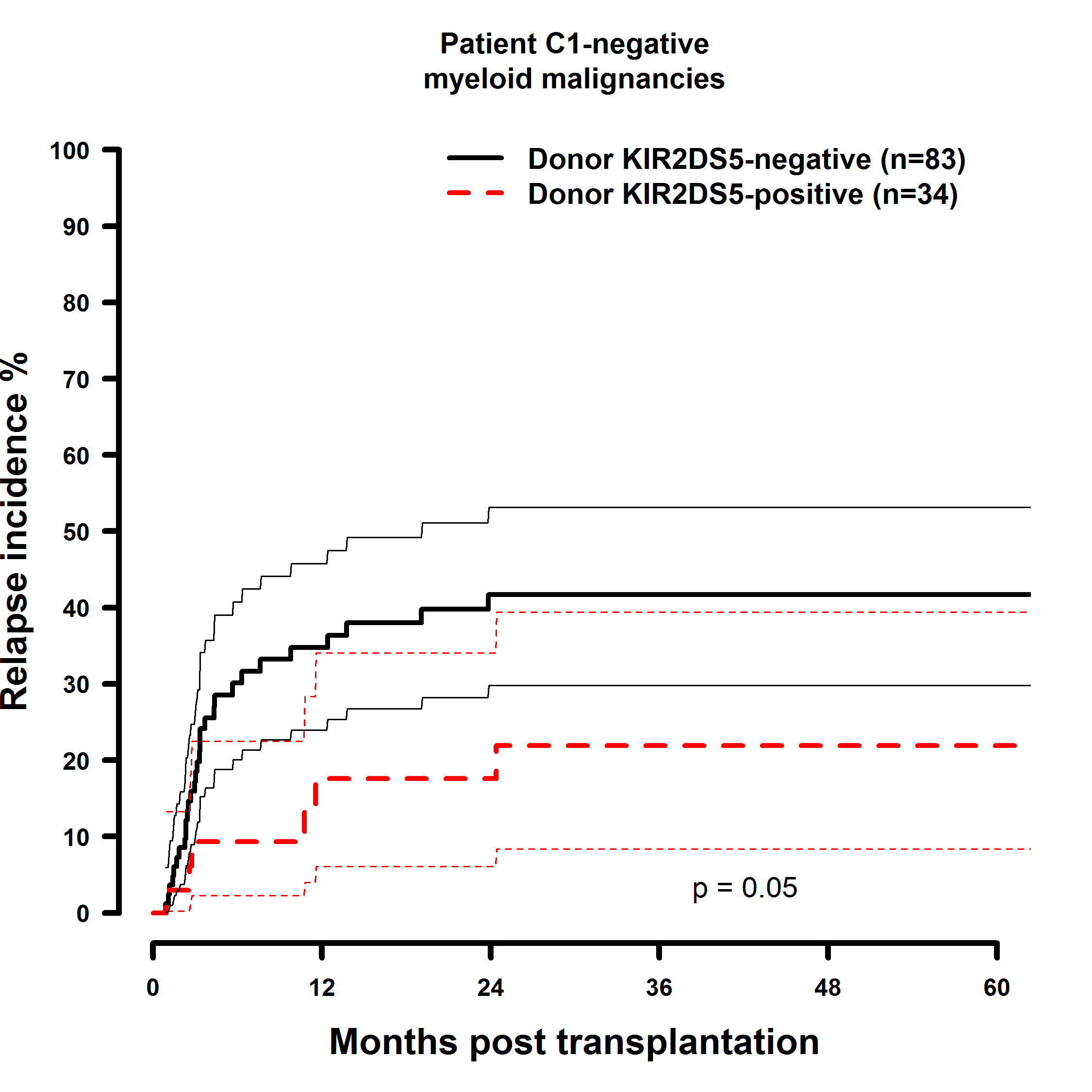

Supplement: S15 Fig — Solid black line: donor KIR2DS5 negative, fine black lines: corresponding confidence intervals. Dashed red line: donor 5 positive, fine red lines: corresponding confidence intervals. Donor KIR2DS5-negative (n = 83) vs. donor KIR2D5-positive (n = 34), p = 0.05. (TIFF) [file pone.0169512.s015.tiff]

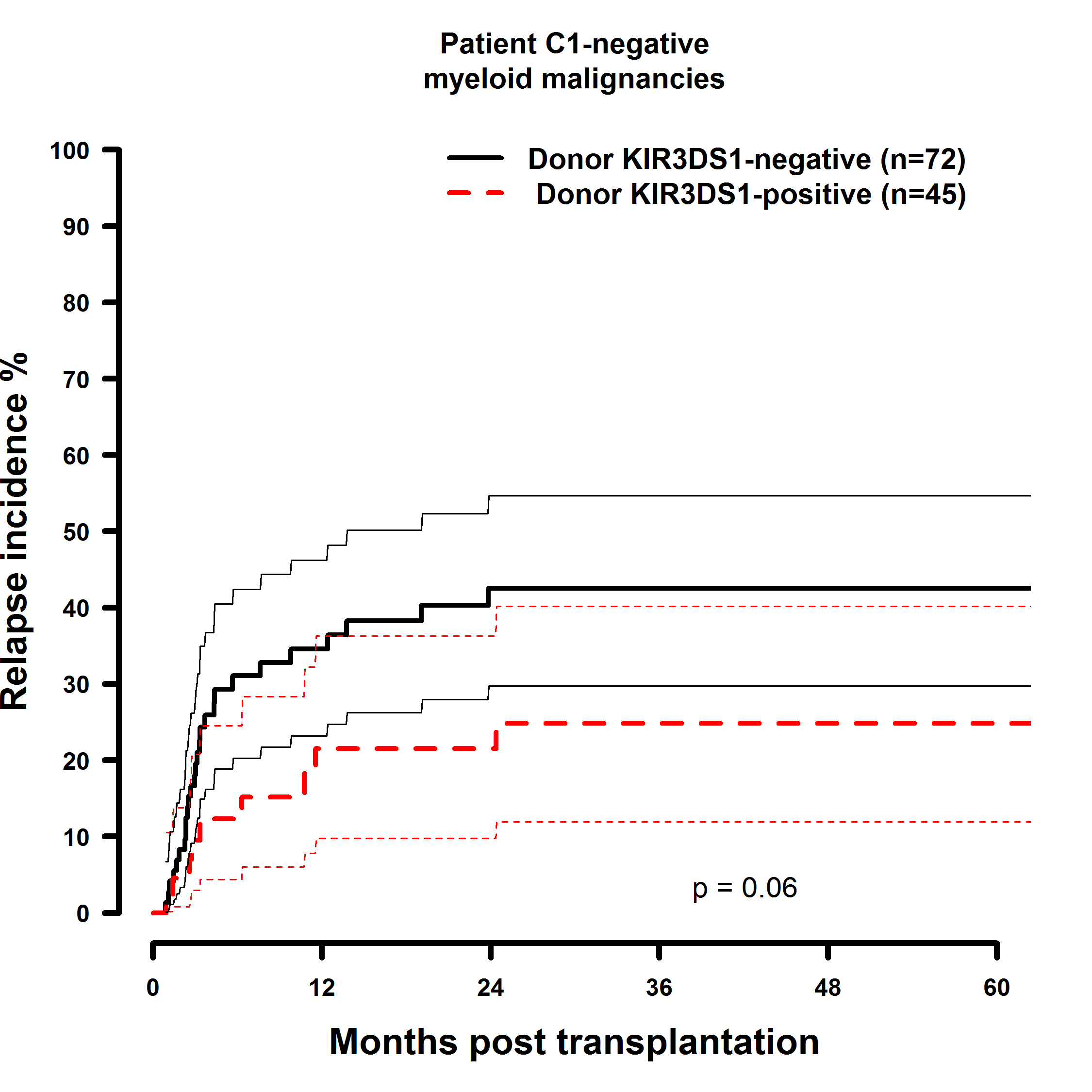

Supplement: S16 Fig — Solid black line: donor KIR3DS1 negative, fine black lines: corresponding confidence intervals. Dashed red line: donor KIR3DS1 positive, fine red lines: corresponding confidence intervals. Donor KIR3DS1-negative (n = 72) vs. donor KIR3DS1-positive (n = 45), p = 0.06. (TIFF) [file pone.0169512.s016.tiff]

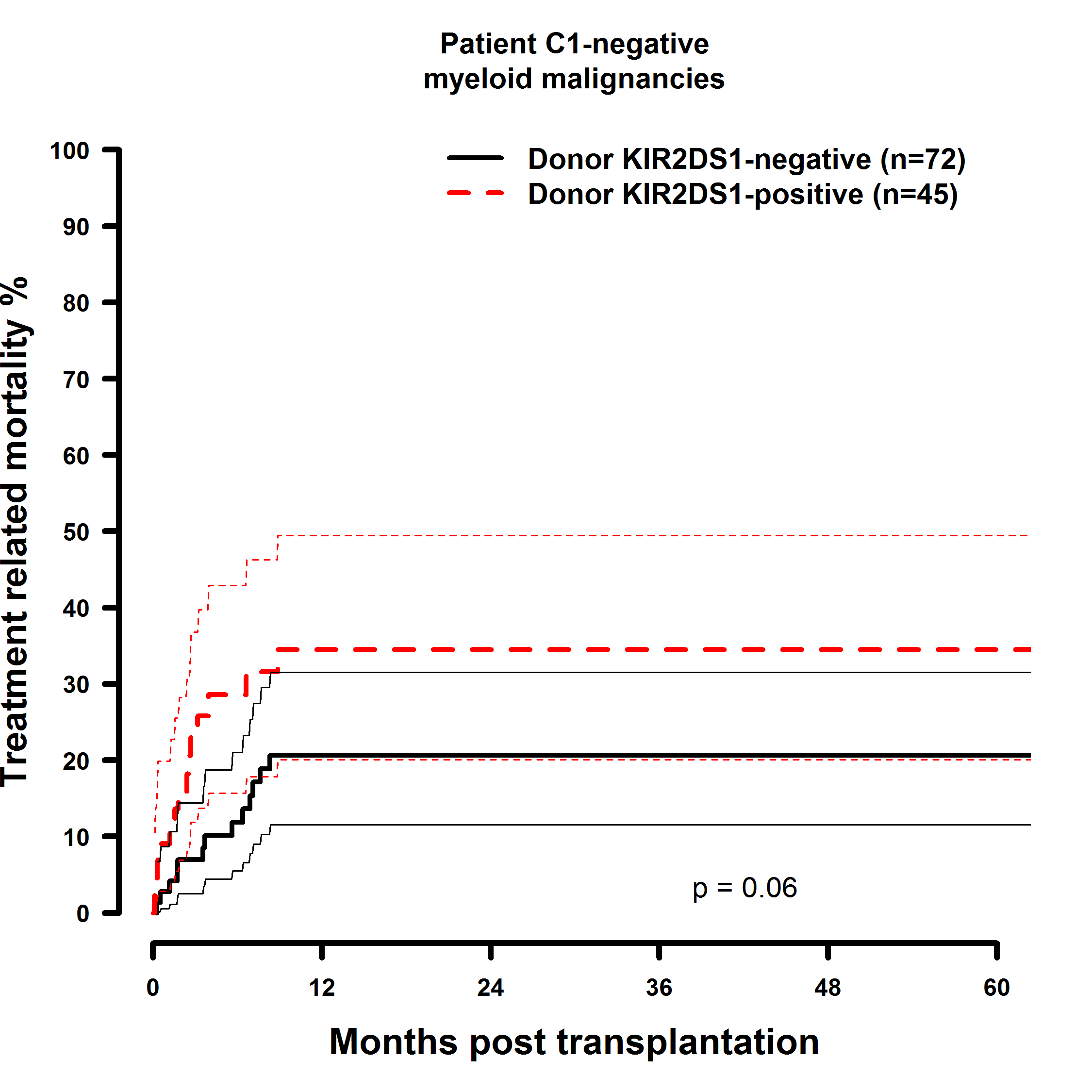

Supplement: S17 Fig — Solid black line: donor KIR2DS1 negative, fine black lines: corresponding confidence intervals. Dashed red line: donor KIR2DS1 positive, fine red lines: corresponding confidence intervals. Donor KIR2DS1-negative (n = 72) vs. donor KIR2DS1-positive (n = 45), p = 0.06. (TIFF) [file pone.0169512.s017.tiff]

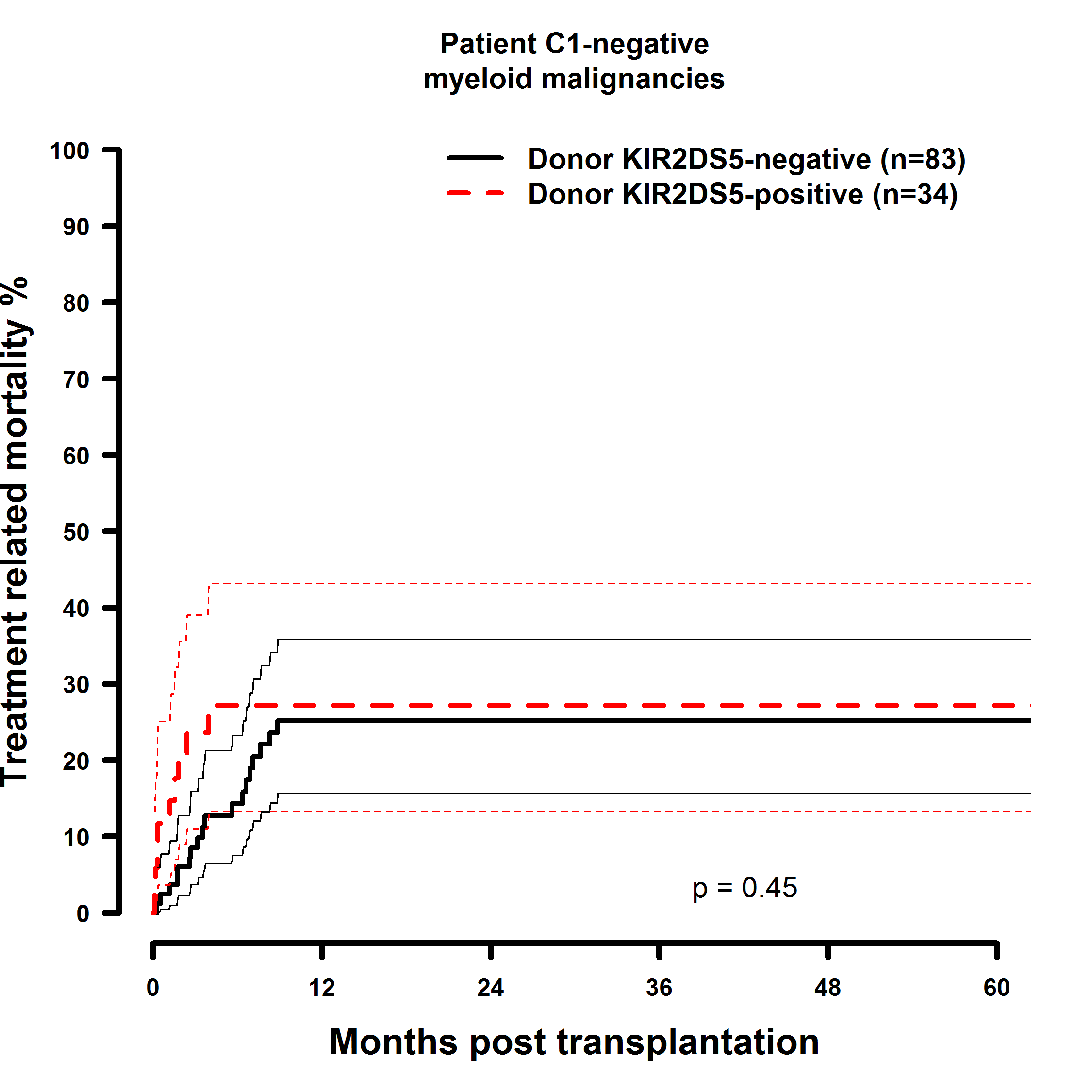

Supplement: S18 Fig — Solid black line: donor KIR2DS5 negative, fine black lines: corresponding confidence intervals. Dashed red line: donor KIR2DS5 positive, fine red lines: corresponding confidence intervals. Donor KIR2DS5-negative (n = 83) vs. donor KIR2D5-positive (n = 34), p = 0.45. (TIFF) [file pone.0169512.s018.tiff]

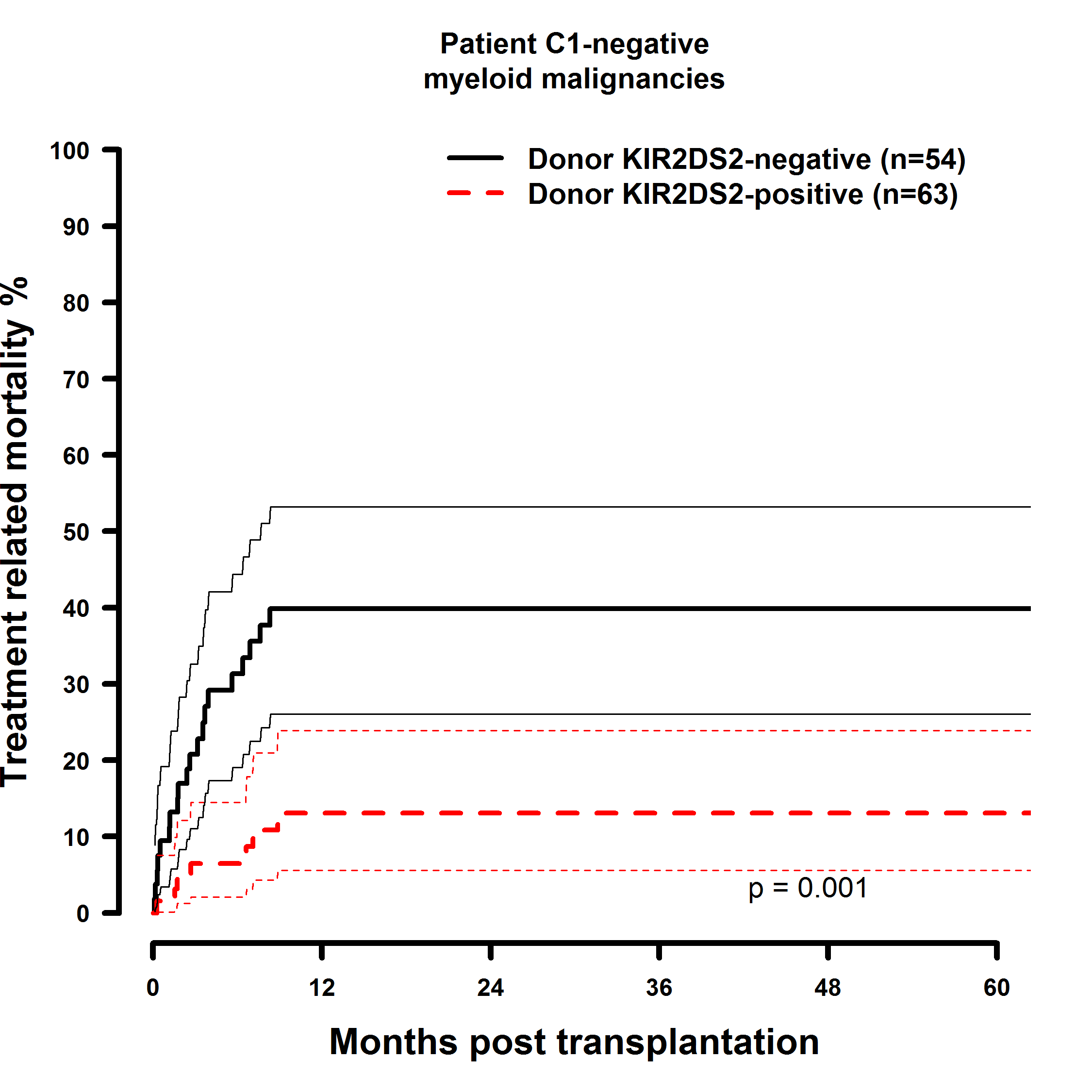

Supplement: S19 Fig — Solid black line: donor KIR2DS2 negative, fine black lines: corresponding confidence intervals. Dashed red line: donor KIR2DS2 positive, fine red lines: corresponding confidence intervals. Donor KIR2DS2-negative (n = 54) vs. donor KIR2DS2-positive (n = 63), p = 0.001. (TIFF) [file pone.0169512.s019.tiff]

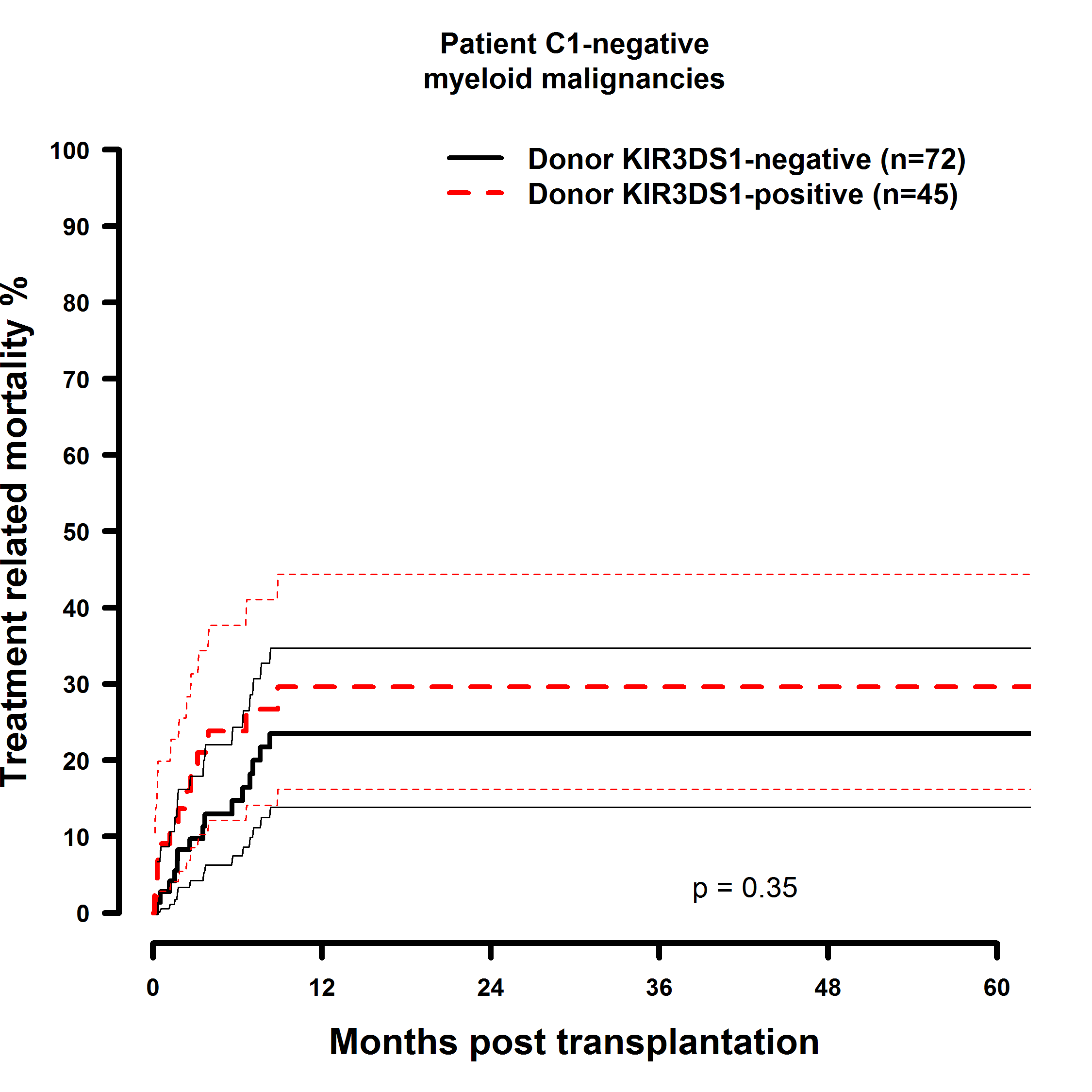

Supplement: S20 Fig — Solid black line: donor KIR3DS1 negative, fine black lines: corresponding confidence intervals. Dashed red line: donor KIR3DS1 positive, fine red lines: corresponding confidence intervals. Donor KIR3DS1-negative (n = 72) vs. donor KIR3DS1-positive (n = 45), p = 0.35. (TIFF) [file pone.0169512.s020.tiff]
