# Supplementary material for: Impact of Donor Activating KIR Genes on HSCT Outcome in C1-Ligand Negative Myeloid Disease Patients Transplanted with Unrelated Donors—A Retrospective Study
Source: PLoS One. 2017 Jan 20;12(1):e0169512. doi: 10.1371/journal.pone.0169512 (PMC5249182; doi:10.1371/journal.pone.0169512)
Supplement: S1 Table — (DOCX) [file pone.0169512.s021.docx]

**S21 Table: Effects of clinical predictors on OS (9/10 KIR2DS2 analysis). Only predictors which reached statistical significance or show a trend are shown.**

|  | **HR** | **95 % CI** | **p** |
| --- | --- | --- | --- |
| **Age (per life year increase)** | 1.05 | 1.00 - 1.10 | 0.04 |
| **Donor KIR2DS2** |  |  |  |
| negative | 1.00 |  |  |
| positive | 0.25 | 0.08 - 0.71 | 0.01 |
| **Conditioning regimen** |  |  |  |
| Myeloablative | 1.00 |  |  |
| Reduced intensity | 3.95 | 1.31 - 11.94 | 0.01 |
