# Supplementary material for: Impact of Donor Activating KIR Genes on HSCT Outcome in C1-Ligand Negative Myeloid Disease Patients Transplanted with Unrelated Donors—A Retrospective Study
Source: PLoS One. 2017 Jan 20;12(1):e0169512. doi: 10.1371/journal.pone.0169512 (PMC5249182; doi:10.1371/journal.pone.0169512)
Supplement: S3 Table — (DOCX) [file pone.0169512.s023.docx]

**S23 Table: Effects of clinical predictors on RI (KIR2DS5 analysis). Only predictors which reached statistical significance or show a trend are shown.**

|  | **HR** | **95 % CI** | **p** |
| --- | --- | --- | --- |
| **Donor KIR2DS5** |  |  |  |
| negative | 1.00 |  |  |
| positive | 0.31 | 0.12 - 0.81 | 0.017 |
| **Disease stage** |  |  |  |
| Early | 1.00 |  |  |
| Intermediate | 2.17 | 0.61 - 7.78 | 0.23 |
| Advanced | 2.22 | 0.89 - 5.56 | 0.09 |
| **Donor source** |  |  |  |
| International | 1.00 |  |  |
| National (german) | 0.18 | 0.07 - 0.47 | < 0.001 |
| **Conditioning regimen** |  |  |  |
| Myeloablative | 1.00 |  |  |
| Reduced intensity | 3.06 | 1.35 - 6.92 | 0.007 |
