# Supplementary material for: Impact of Donor Activating KIR Genes on HSCT Outcome in C1-Ligand Negative Myeloid Disease Patients Transplanted with Unrelated Donors—A Retrospective Study
Source: PLoS One. 2017 Jan 20;12(1):e0169512. doi: 10.1371/journal.pone.0169512 (PMC5249182; doi:10.1371/journal.pone.0169512)
Supplement: S4 Table — (DOCX) [file pone.0169512.s024.docx]

**S24 Table: Effects of clinical predictors on RI (KIR3DS1 analysis). Only predictors which reached statistical significance or show a strong trend are shown.**

|  | **HR** | **95 % CI** | **p** |
| --- | --- | --- | --- |
| **Donor KIR3DS1** |  |  |  |
| negative | 1.00 |  |  |
| positive | 0.46 | 0.21 - 1.04 | 0.06 |
| **Disease stage** |  |  |  |
| Early | 1.00 |  |  |
| Intermediate | 1.83 | 0.58 - 5.78 | 0.30 |
| Advanced | 2.28 | 0.93 - 5.57 | 0.07 |
| **Donor source** |  |  |  |
| International | 1.00 |  |  |
| National (german) | 0.20 | 0.08 - 0.50 | < 0.001 |
| **Conditioning regimen** |  |  |  |
| Myeloablative | 1.00 |  |  |
| Reduced intensity | 2.97 | 1.31 - 6.71 | 0.009 |
